# High-throughput chemogenetic drug screening reveals PKC-RhoA/PKN as a targetable signaling vulnerability in *GNAQ*-driven uveal melanoma

**DOI:** 10.1016/j.xcrm.2023.101244

**Published:** 2023-10-18

**Authors:** Nadia Arang, Simone Lubrano, Michele Ceribelli, Damiano C. Rigiracciolo, Robert Saddawi-Konefka, Farhoud Faraji, Sydney I. Ramirez, Daehwan Kim, Frances A. Tosto, Erica Stevenson, Yuan Zhou, Zhiyong Wang, Julius Bogomolovas, Alfredo A. Molinolo, Danielle L. Swaney, Nevan J. Krogan, Jing Yang, Silvia Coma, Jonathan A. Pachter, Andrew E. Aplin, Dario R. Alessi, Craig J. Thomas, J. Silvio Gutkind

**Affiliations:** 1 Moores Cancer Center, University of California San Diego, La Jolla, CA 92093, USA; 2 Biomedical Sciences Graduate Program, University of California San Diego, La Jolla, CA 92093, USA; 3 Department of Pharmacology, School of Medicine, University of California San Diego, La Jolla, CA 92093, USA; 4 Division of Preclinical Innovation, National Center for Advancing Translational Sciences, National Institutes of Health, Rockville, MD 20850, USA; 5 Department of Pharmacy, University of Pisa, Pisa, Italy; 6 School of Medicine, University of California San Diego, La Jolla, CA 92093, USA; 7 Quantitative Biosciences Institute (QBI), University of California San Francisco, San Francisco, CA 94158, USA; 8 J. David Gladstone Institutes, San Francisco, CA 94158, USA; 9 Department of Cellular and Molecular Pharmacology, University of California San Francisco, San Francisco, CA 94158, USA; 10 Department of Pediatrics, University of California San Diego, La Jolla, CA 92093, USA; 11 Verastem Oncology, Needham, MA 02494, USA; 12 Department of Cancer Biology, Thomas Jefferson University, Philadelphia, PA 19107, USA; 13 Medical Research Council (MRC) Protein Phosphorylation and Ubiquitylation Unit, School of Life Sciences, University of Dundee, Dundee DD1 5EH, UK

**Keywords:** melanoma, GNAQ, chemogenetic drug screening, PKC, PKN/PRK, FAK, synthetic lethality, combination therapy, precision medicine

## Abstract

Uveal melanoma (UM) is the most prevalent cancer of the eye in adults, driven by activating mutation of *GNAQ/GNA11*; however, there are limited therapies against UM and metastatic UM (mUM). Here, we perform a high-throughput chemogenetic drug screen in *GNAQ*-mutant UM contrasted with *BRAF*-mutant cutaneous melanoma, defining the druggable landscape of these distinct melanoma subtypes. Across all compounds, darovasertib demonstrates the highest preferential activity against UM. Our investigation reveals that darovasertib potently inhibits PKC as well as PKN/PRK, an AGC kinase family that is part of the “*dark kinome.*” We find that downstream of the Gαq-RhoA signaling axis, PKN converges with ROCK to control FAK, a mediator of non-canonical Gαq-driven signaling. Strikingly, darovasertib synergizes with FAK inhibitors to halt UM growth and promote cytotoxic cell death *in vitro* and in preclinical metastatic mouse models, thus exposing a signaling vulnerability that can be exploited as a multimodal precision therapy against mUM.

## Highlights

• A chemogenetic drug screen in uveal melanoma reveals darovasertib as a top hit
• Darovasertib is a dual inhibitor of PKC and PRK/PKN
• Downstream of mutant Gαq, darovasertib blocks ERK and diminishes FAK activity
• Darovasertib + FAKi act synergistically in preclinical uveal melanoma models

## Graphical abstract

**Figure:**
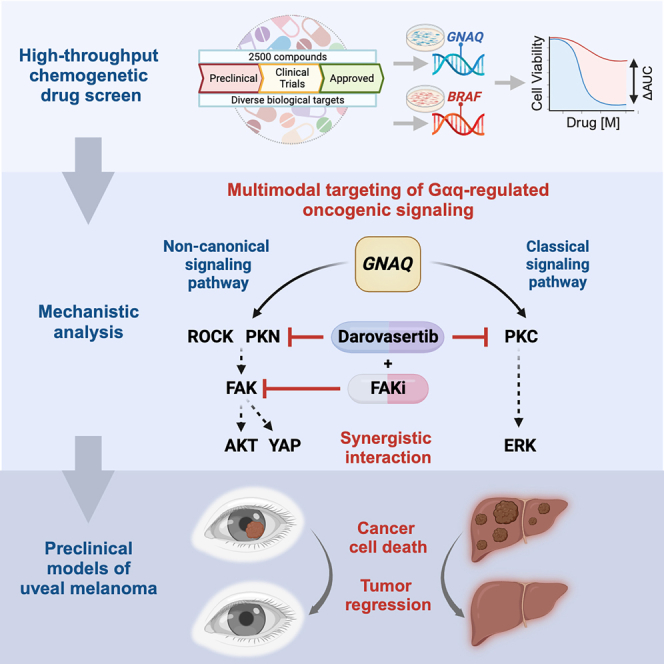

## Teaser

Arang et al. define the druggable landscape of uveal melanoma and identify darovasertib as the top hit. Darovsertib acts as a dual PKC and PRK/PKN inhibitor, thereby blocking ERK and diminishing FAK activity. Darovasertib combined with FAK inhibition is highly synergistic in preclinical models of primary and metastatic uveal melanoma.

## Introduction

In the current era of precision medicine, molecular-targeted therapies have transformed the standard of care and clinical outcomes for numerous cancer types. Examples of success including imatinib for BCR-ABL-driven chronic myeloid leukemia,^1^ and erlotinib against non-small cell lung cancer,^2^ this approach takes advantage of cancer-specific oncogene addiction that can serve as actionable therapeutic targets. This paradigm has proven to be particularly true for cancer types with well-defined cancer-driving genetic alterations, in which the integration of genomic data to functional signaling events can be readily translated into targetable molecular vulnerabilities.

However, in spite of their clearly identifiable oncogenic drivers, we still lack effective targeted therapies for many human malignancies. This includes uveal melanoma (UM), the most common intraocular malignancy in adults, and the second most frequent melanoma site after skin cutaneous melanoma (SKCM).^3^ UM is unique among adult cancers with one of the lowest mutation burdens across all cancers in The Cancer Genome Atlas.^3^ Whereas most SKCM typically possess characteristic *BRAF* or *NRAS* mutations, UM is driven by aberrant activation of the Gαq pathway, with >95% of patients harboring gain-of-function mutation of *GNAQ/GNA11*, encoding the Gαq subunit family of heterotrimeric G proteins, and rendering them as driver oncogenes.^3^^,^^4^^,^^5^ Patients lacking *GNAQ/11* mutations typically possess mutation and subsequent aberrant activation of *CYSLTR2*, a Gαq-coupled GPCR.^3^^,^^6^ Roughly 50% of UM patients progress to metastatic UM (mUM), which is associated with loss of function in BAP1 and is highly refractory to current therapies with a median survival of approximately 1 year.^7^^,^^8^^,^^9^^,^^10^

Gαq and Gαq-coupled GPCRs have been long implicated as drivers of neoplastic growth, involved in numerous human malignancies, including UM.^4^^,^^5^^,^^11^^,^^12^^,^^13^ However, despite the clean genetic landscape of UM, there are limited effective targeted therapies currently available. This is likely due in part to the complexity of the mechanisms by which Gαq and Gαq-coupled GPCRs promote aberrant cell proliferation.^14^^,^^15^^,^^16^ Canonically, Gαq signals through PLCβ, initiating the generation of second-messenger systems that lead to the activation of the MEK/ERK cascade via RasGEFs such as RASGRP3.^17^^,^^18^ However, clinical efforts aimed toward inhibition of MEK/ERK signaling using agents including trametinib and selumetinib have not demonstrated a significant clinical benefit or improvement in overall patient survival.^19^^,^^20^^,^^21^ In this context, we have shown that parallel to the canonical signaling axis, Gαq controls a non-canonical oncogenic signaling axis through the RhoGEF TRIO.^22^ This leads to the activation of FAK, which then promotes the aberrant activation of YAP and PI3K pathways to drive tumor growth.^14^^,^^23^

Moreover, we and others have shown that direct inhibition of Gαq/11 using agents such as FR900359, a cyclic depsipeptide, effectively block Gαq function and decrease UM growth, but the centrality of Gαq to essential physiological processes including neurotransmission, cardiac function, and vasculogenesis, may pose a significant challenge toward the development of safe agents targeting Gαq for the treatment of UM and other Gαq-driven malignancies in the clinic.^24^^,^^25^ Of interest, Tebentafusp, a bispecific fusion protein, has been recently approved in unresectable or mUM patients; however, only 50% of the patient population is eligible based on HLA haplotype restriction, and responses remain limited with a 9% objective response rate.^26^^,^^27^^,^^28^ Taken together, there is a critical and urgent need for novel therapeutic strategies against mUM and advanced primary UM cases.

In this regard, functional genomics approaches have served as highly valuable tools for the identification of molecular targets for multiple cancer types.^29^^,^^30^ However, our ability to translate cancer cell dependencies to the clinic is often not limited to the discovery of novel gene candidates, but rather restricted by the toolbox of approved, or soon-to-be approved pharmacological agents at our disposal. The limited genetic aberrancies in UM and its defined oncogenic signaling drivers, coupled with the lack of FDA-approved targeted therapies provided a unique opportunity to use a network chemical biology-based approach to identify pharmacologically tractable UM-specific vulnerabilities that can be readily translated to the clinic.

## Results

### A chemogenetic screen defines the druggable landscape of UM

**Figure 1:**
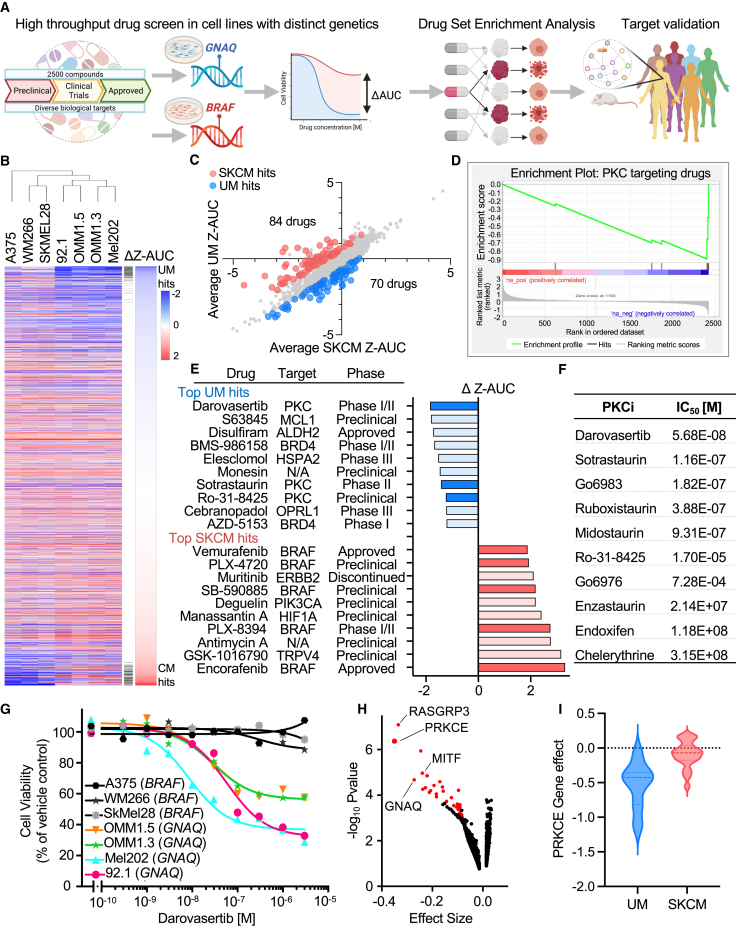
Mechanistic and target-level dependencies enriched in *GNAQ*-mutant UM (A) Schematic of screening pipeline in *GNAQ*-mutant and BRAF-mutant cell lines. Created with Biorender. (B) Heatmap depicting Z-transformed area under the curve (AUC) scores for all cell lines versus MIPE 5.0 compound library. Compounds (rows) were sorted based on the difference in average Z-AUC (ΔZ-AUC) between UM and SKCM cell contexts. Unsupervised hierarchical clustering was performed on cell lines (columns). Context-selective drugs (rows) are marked on the right. (C) Average Z-AUCs for UM plotted against SKCM. Hits identified in the UM context are highlighted in blue and SKCM hits are highlighted in red. (D) Enrichment plot for PKC-targeting drugs in UM cell lines. (E) Top 20 selective drugs ranked by Z-AUC score (UM selective above, SKCM selective below). PKC-targeting drugs are shaded in darker blue, and BRAF-targeting drugs are shaded in darker red. (F) IC_50_ values for all tested PKCi across UM cell lines. (G) Cell viability dose response of darovasertib in all cell lines screened. (H) Two-class comparison of PRKCE CRISPR gene effect plotted against –log10 p value for UM versus SKCM cell lines from DepMap 21Q1 Public Dataset. Genes in red have a q value < 0.05. (I) Gene effect for PRKCE in UM and SKCM cell lines from DepMap 22Q2 Public+Score, Chronos Dataset. See also Tables S1, S2, and S3.

To comprehensively characterize the druggable landscape of *GNAQ*-mutant UM, we performed a high-throughput chemogenetic screen in four genetically distinct *GNAQ*-mutant UM cell-lines, using three *BRAF*-mutant SKCM as compare/contrast controls. Specifically, we used a collection of ∼2,500 mechanistically annotated, oncology-focused agents, greater than 50% of which are in clinical trials or already FDA approved. This library, known as MIPE 5.0, purposely exploits target-level redundancy (multiple inhibitors for key onco-targets), while simultaneously encompassing mechanistic and biological diversity, targeting more than 800 distinct mechanisms of action (Figure 1A).^31^ For each cell line, we performed a full 11-point dose titration for each agent, generating nearly 20,000 dose-response profiles that were used to interrogate the drug-sensitivity landscape of UM and SKCM. We used Z-transformed, area under the curve (Z-AUC) scores as the primary quantitative metric to rank compound activities and classify hits. Importantly, unsupervised hierarchical clustering of drug-activity profiles resulted in the separation of distinct UM and SKCM cell clusters demonstrating strong genotype-driven compound sensitivities (Figure 1B).

Based on the clustering results, we first captured the Z-AUC difference between the two groups to identify dugs with preferential activity against UM (70 UM-specific hits) or SKCM (84 SKCM-specific hits) (Figure 1C; Table S1). We next exploited MIPE 5.0 mechanistic redundancy to systematically identify target-level dependencies in each group (see STAR Methods for details). Our approach revealed a number of target classes that have not yet been extensively explored in the context of UM, including XPO1, CHEK1, and MCL1, and a high representation of epigenetic modifiers including agents targeting HDAC1,6 and BRD2,4 (Tables S2 and S3). PKC-targeting drugs also emerged among the top hits (Figures 1D and 1E; Table S2). Given the relevance of PKC to canonical Gαq signaling, we chose to further investigate the subset of agents targeting PKC from our screen.^18^^,^^32^^,^^33^ Among them, darovasertib (LXS-196), a PKC inhibitor (PKCi) under current clinical investigation for the treatment of UM,^34^ exhibited the most differential Z-AUC score, and lowest IC_50_ across all PKCi tested (Figures 1E and 1F). Aligned with the basis of our screen, dose-response curves of darovasertib demonstrated strong UM-specific activity in comparison with SKCM cell viability (Figure 1G). As an independent target validation, PKCε (PRKCE) is among the top cell essential genes in UM, with a significantly stronger cell-dependency score in UM compared with SKCM cell lines in the Depmap Portal as determined by whole-genome-wide CRISPR screening efforts (Figures 1H and 1I). Taken together, this convergence of genetic and pharmacological data establishes PKC as a critical survival node in UM. However, PKC inhibition has been tested in mUM in the clinic, with limited responses.^35^ In this regard, we noticed that darovasertib was more effective than other PKCi evaluated, which raised the possibility that this agent may be more potent regarding target inhibition, or harbor other yet to be identified properties leading to an increased response in UM cells.

### Multi-targeted activity of darovasertib underlies its potency in UM

**Figure 2:**
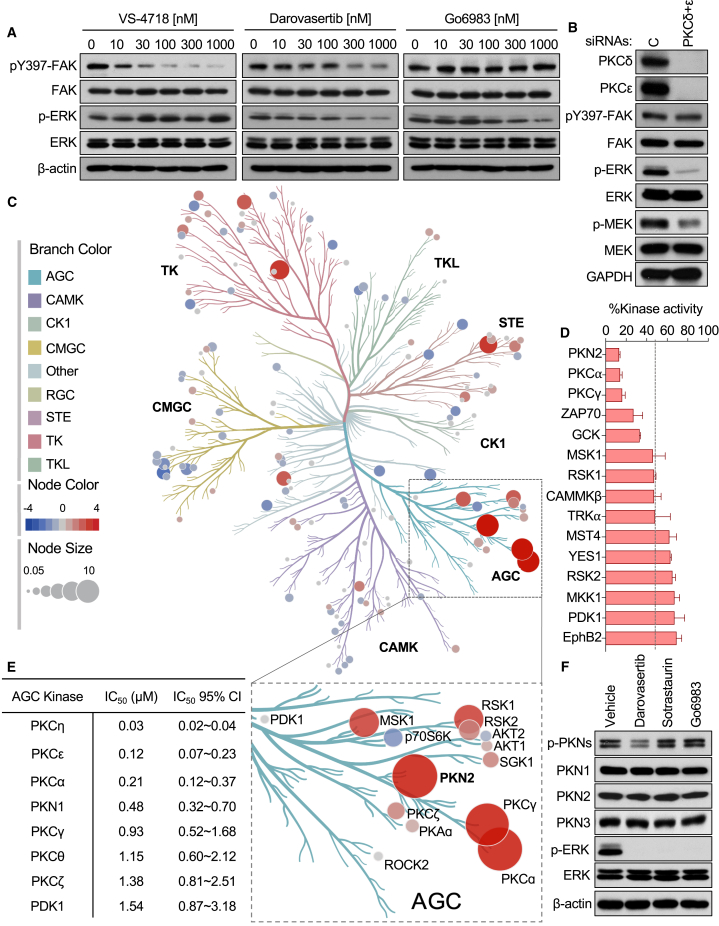
Multi-targeted activity of darovasertib underlies its potency in UM (A) Dose-dependent effects on phosphorylated FAK and ERK in 92.1 UM cells in response to treatment with VS-4718, darovasertib, or Go6983 for 2 h. (B) Impact of siRNA-mediated knockdown of PKCδ+ε on phosphorylated FAK, ERK, and MEK in 92.1 UM cells. (C) Kinome profiling of darovasertib. Node size and color indicate degree of kinase inhibition in response to 1 μM darovasertib, with reduction in kinase activity as red, and increase in kinase activity as blue. The figure was generated using Coral.^75^ (D) Percent kinase activity remaining after treatment with 1 μM darovasertib for the top 15 kinases with highest inhibition. (E) IC_50_ and 95% CI of darovasertib on recombinant enzymes for a sub-panel of AGC kinases. (F) Phosphorylation of PKNs and ERK in response to treatment with a panel of PKCi: 1 μM darovasertib, 1 μM sotrastaurin, or 1 μM Go6983 for 1 h in 92.1 UM cells.

We next investigated whether darovasertib exerts unique activities that may help explain its increased activity in UM compared with all other compounds tested in our screen. We first examined its signaling inhibition profile against the two major described signaling axes downstream of Gαq, using pERK as a surrogate of inhibition of canonical Gαq-driven signaling through PLCβ, and pFAK as a measure of inhibition of non-canonical signaling downstream of Gαq through TRIO and RhoA.^14^^,^^15^^,^^22^ We compared the activity of darovasertib against Go6983, a broad-spectrum highly selective PKCi, and VS-4718, a highly specific FAK inhibitor (FAKi) (Figure 2A).^36^^,^^37^ We observed that both PKCi tested inhibited pERK at similar levels. However, we found that darovasertib partially diminished pFAK, whereas Go6983 treatment had no effect on pFAK (Figure 2A). As a control, FR900359 (FR), an inhibitor of Gαq, potently abrogated all Gαq-driven signaling (Figure S1A). This distinct activity of darovasertib led us to ask whether this agent may help us identify a new mechanism whereby PKC could control FAK activity. To test this, we performed siRNA-mediated knockdown (KD) of PKCδ and PKCε, the primary PKC isoforms described to be critical in UM.^17^ Aligned with our gene essentiality data (Figures 1H and 1I), we found that, while PKC KD led to a potent decrease in pERK, it did not affect pFAK (Figure 2B). This suggested that the ability of darovasertib to reduce pFAK is not concordant with inhibition of PKC, but that instead it may involve additional yet to be elucidated mechanisms.

To explore this possibility, we performed a kinome-wide screen testing the capacity of darovasertib to inhibit enzymatic activity against 140 kinases using a highly sensitive radioactive filter binding assay that provides a direct measure of activity^38^^,^^39^ (Figure 2C). Among the top kinases with greater than 75% activity inhibition over control included PKC isozymes, as expected (Figures 2C and 2D). However, of interest, the more strongly inhibited kinase was PKN2, a member of the PKN (also known as PRK) kinase subfamily (Figures 2C and 2D). While they belong to the same kinase superfamily, PKCs and PKNs (consisting of three members PKN1, 2, and 3) have distinct N-terminal regulatory regions and diverge in their activation mechanisms.^40^ Namely, in contrast to the PKCs, the PKNs are a family of Rho-responsive kinases, and have been shown to be involved in numerous functions, including actin cytoskeleton remodeling, cell migration, and cell-cycle regulation.^40^^,^^41^^,^^42^^,^^43^^,^^44^^,^^45^ To complement our kinome selectivity screen, we next performed a detailed analysis of darovasertib activity against a selection of AGC family kinases using recombinant proteins and found strong activity against novel and conventional classes of PKCs in addition to PKN1 (Figure 2E). Aligned with the results of our screen, we found that darovasertib treatment potently decreased the accumulation of the phosphorylated, active form of PKN (pPKN); however, treatment with Go6983 or sotrastaurin, the latter a clinical PKCi that failed to demonstrate significant clinical benefit in mUM patients^35^ did not result in a change in pPKN levels (Figure 2F).

### PKN converges with ROCK to control FAK downstream of the Gαq-RhoA signaling axis

**Figure 3:**
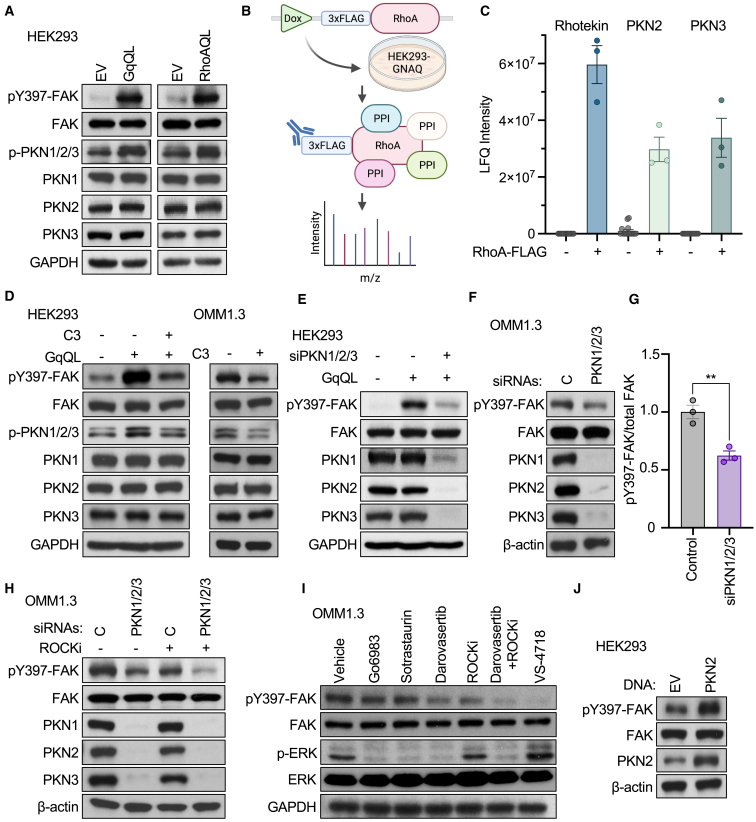
PKN converges with ROCK to control FAK downstream of the Gαq-RhoA signaling axis (A) Phosphorylation of FAK and PKNs in HEK293 cells transfected with empty vector, Gαq-QL, or RhoA-QL active mutants. (B) Schematic of affinity purification mass spectrometry pipeline used to identify RhoA and its associated protein binding partners after doxycycline-inducible FLAG-tagged RhoA was expressed in HEK293 cells with stable overexpression of Gαq. Created with Biorender. (C) Label-free quantification (LFQ) intensity of RhoA binding partners after RhoA expression was induced by 1 μM doxycycline treatment for 42 h. (D) Phosphorylation of FAK and PKNs in response to expression of Gαq-QL alone or in combination with RhoA blockade using 2 μg/mL C3 toxin for 16 h in HEK293 cells (left) or UM cells (right). (E) Phosphorylation of FAK after expression of Gαq-QL alone or in combination with siRNA-mediated knockdown of PKNs in HEK293 cells. (F) Phosphorylation of FAK in UM cells in response to siRNA-mediated knockdown of PKNs. (G) Quantification of pFAK signal normalized to total FAK levels from (F) (mean ± SEM, n = 3). (H) Phosphorylation of FAK in UM cells in response to siRNA-mediated knockdown of PKNs, alone or in combination with ROCK inhibition using 10 μM ROCKi (Y-27632) for 1 h. (I) Phosphorylation of FAK and ERK in UM cells in response to a panel of inhibitors, all used at 1 μM for 1 h. (J) FAK phosphorylation in response to overexpression of PKN2 in HEK293 cells.

We next sought to determine the mechanism by which PKN controls FAK activity. We have previously shown that FAK acts as a central mediator of oncogenic signaling in UM by transducing signaling that is driven by mutant Gαq through the RhoA-ROCK signaling pathway.^14^ To first understand the impact of a parallel RhoA-PKN signaling axis on controlling FAK activity, we expressed constitutively active mutants of Gαq (GαqQL) and RhoA (RhoAQL) in HEK293 cells as a widely used experimental cellular system. Immunoblotting against total and phosphorylated forms of FAK and PKN revealed that activation of Gαq or its downstream RhoA-initiated signaling mechanisms led to an increase in active phosphorylated FAK and PKN (Figure 3A). PKN has also been shown to be activated in response to binding to RhoA. To test this in an unbiased manner, and in the context of Gαq-driven signaling, we performed affinity purification mass spectrometry after doxycycline-inducible expression of an FLAG-tagged RhoA in Gαq-expressing HEK293 cells (Figure 3B). Indeed, upon expression of RhoA-FLAG, we observed robust binding of RhoA to Rhotekin, a canonical RhoA-activated protein, as well as PKN2 and PKN3 (Figure 3C).^46^ Aligned with this, blockade of RhoA activity using C3 toxin, a potent and specific inhibitor of RhoA, abrogated the increase of pFAK and pPKN in response to pathway activation by GαqQL (Figure 3D, left). Similarly, treatment of UM cells harboring Gαq mutations endogenously with C3 toxin decreased pFAK and pPKN (Figure 3D, right).

We next assessed the impact of PKN on FAK activity, and found that, in both HEK293 cells transfected with GαqQL and in UM cells, that knockdown of PKN isoforms, which are broadly expressed in UM cells, resulted in a significant decrease in pFAK levels (Figures 3E, 3F, 3G, S2A, and S2B). Based on these results, we hypothesized that Gαq non-canonical signaling branches at the level of RhoA into ROCK- and PKN-mediated signaling, converging on the promotion of FAK activity. To test this, we examined pFAK levels in response to the combination of PKN and ROCK inhibition. Independently, inhibition of PKN either using siRNA-mediated knockdown (Figure 3H) or by darovasertib (Figure 3I) resulted in a partial decrease in pFAK levels. In contrast, inhibition of PKN concomitant with ROCK inhibition potently decreased pFAK with similar efficiency as VS-4718, suggesting that RhoA controls FAK via two distinct ROCK- and PKN-mediated signaling axes (Figures 3H and 3I). Finally, to assess if PKN can directly promote the activity of FAK, we overexpressed PKN2 in HEK293 cells, and observed a marked increase in pFAK (Figure 3J). These results suggest that PKN is a component of the Gαq-regulated signaling circuitry in UM, and that, together with ROCK, PKN converges on the promotion of FAK activity. This may in turn suggest that darovasertib inhibits FAK activity by blocking PKN independently of its activity on PKC.

### Impact of darovasertib on the FAK/YAP signaling axis in UM

**Figure 4:**
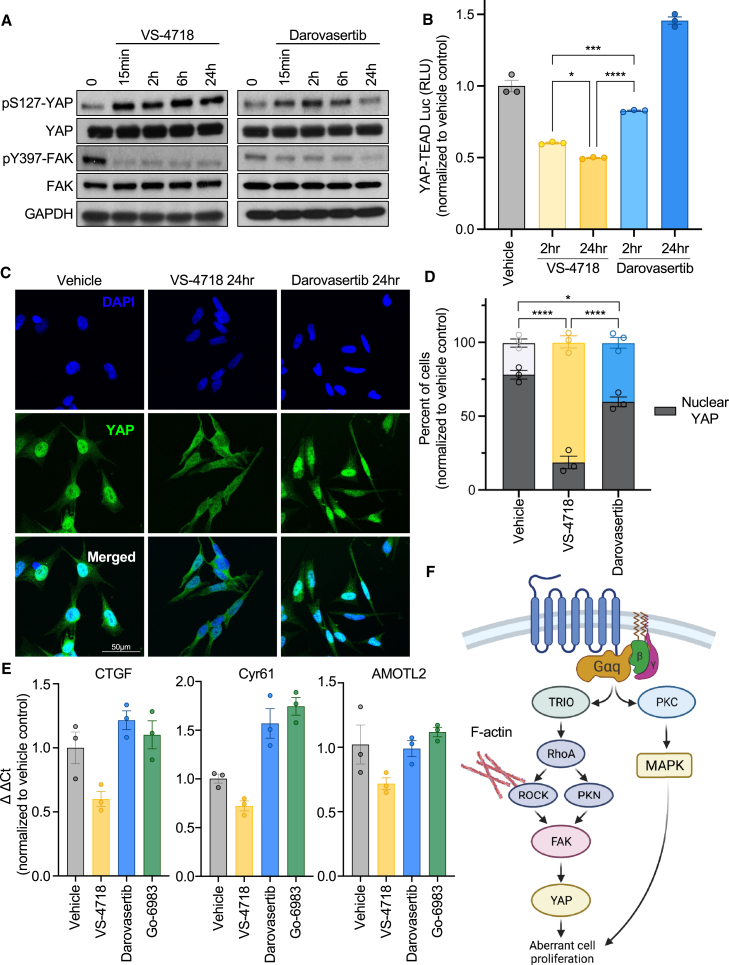
Impact of darovasertib on the FAK/YAP signaling axis in UM (A) YAP and FAK phosphorylation in response to 1 μM VS-4718 or 1 μM darovasertib over a time course in OMM1.3 UM cells. (B) YAP/TAZ luciferase reporter assay after 1 μM VS-4718 or 1 μM darovasertib treatment for 2 or 24 h in UM cells (mean ± SEM, n = 3) in 92.1 UM cells. (C) Monitoring of endogenous YAP subcellular localization by immunofluorescent staining (green), and DAPI staining for nuclear DNA (blue) in UM cells after 1 μM VS-4718 or 1 μM darovasertib treatment for 24 h, vehicle treatment was used as a control in OMM1.3 UM cells. Scale bar, 50 μm. (D) Quantification of (C) showing fraction of cells with nuclear YAP localization in gray, and cytoplasmic fraction in color (vehicle as black, VS-4718 as gold, darovasertib as blue) (mean ± SEM, n = 3). (E) mRNA expression of YAP target genes (CTGF, CYR61, AMOTL2) in response to 1 μM VS-4718, 1 μM darovasertib, and 1 μM Go6983 for 24 h (mean ± SEM, n = 3) in 92.1 UM cells. (F) Schematic depicting the non-canonical signaling pathway regulating FAK activation by Gαq. Signaling downstream of RhoA is co-regulated by ROCK and PKN, converging on FAK activity. Created with Biorender.

FAK has been shown to play a significant functional role in promoting tumor growth in UM by controlling YAP activity.^14^ Thus, we asked if the indirect inhibition of FAK through PKN by darovasertib could control YAP as potently as direct FAK inhibition. As a tumor promoter, YAP is under negative regulation by the Hippo kinase cascade, namely by its phosphorylation on multiple sites, including S127 by the LATS1/2 kinases, which promotes its cytoplasmic retention and subsequent degradation.^47^^,^^48^ Thus, we first assessed levels of YAP pS127 in response to FAK inhibition directly or via darovasertib-mediated inhibition of PKN. Notably, while direct inhibition of FAK by VS-7418 promoted a potent and sustained increase in pS127 YAP, darovasertib transiently increased YAP phosphorylation that reverted to baseline by 24 h (Figure 4A). YAP is a transcriptional co-activator, which, together with the TEAD family of transcription factors, controls complex pro-growth transcriptional programs.^48^ To profile the functional impact of darovasertib on YAP activity, we first assessed YAP transcriptional activity using a YAP/TEAD luciferase reporter. We found that, aligned with our prior observations, while VS-4718 and darovasertib both had early effects on inhibiting YAP/TEAD activity, the observed inhibition was lost in the darovasertib condition over time (Figure 4B). To complement this, we assessed YAP nuclear localization by immunofluorescence in UM cells, which express high levels of nuclear YAP at baseline conditions. Aligned with the sustained increase in pS127 YAP after direct FAK inhibition, we observed significant reduction in nuclear-localized YAP, indicative of its suppression (Figures 4C and 4D). In contrast, darovasertib treatment resulted in a partial reduction in YAP nuclear localization (Figures 4C and 4D). Finally, we assessed levels of canonical YAP transcriptional targets. While VS-4718 treatment led to a decrease in mRNA levels of CTGF, CYR61, and AMOTL2 as reported previously,^14^ darovasertib treatment did not result in a decrease of gene expression, similar to treatment with Go6983 (Figure 4E). While these data suggest that darovasertib interferes with Gαq-driven signaling by dual inhibition of PKC and PKN, it is insufficient to control FAK/YAP signaling fully (Figure 4F), which led us to explore the effects of darovasertib drug combination *in vitro* and in *in vivo* UM models of disease (see below).

### High-throughput and targeted combinatorial screens reveal that FAKi and darovasertib are synergistic in UM *in vitro*

**Figure 5:**
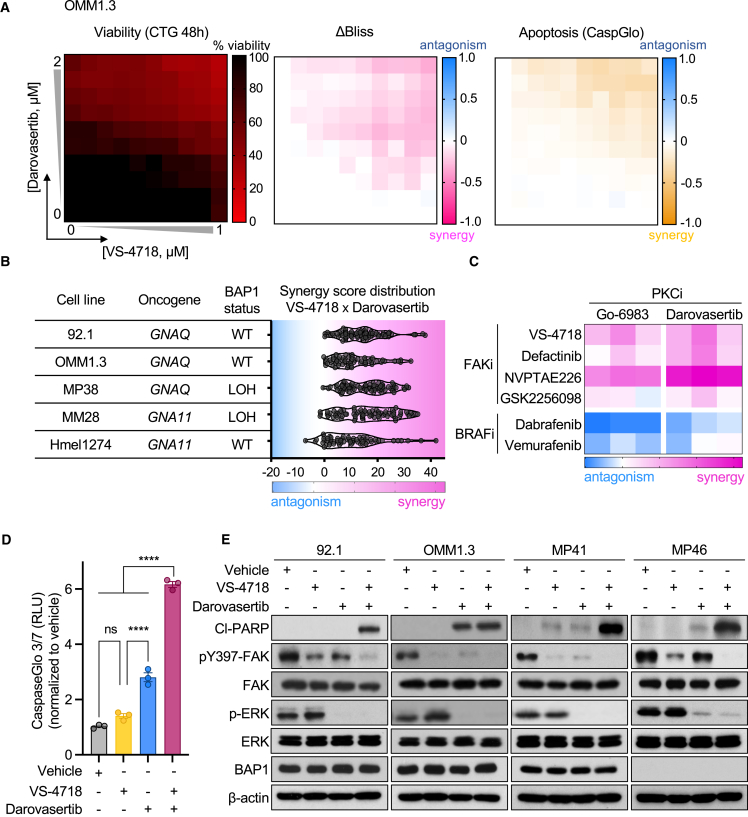
High-throughput and targeted combinatorial screens reveal that FAKi and darovasertib are synergistic in UM *in vitro* (A) Assessment of synergy in UM cells treated with a combination of VS-4718 and darovasertib. Cell viability was measured using CellTiter Glo assay 48 h after treatment (left). Combination index (CI) was determined using the ΔBliss method (CI < 1 synergism, CI = 1 additivity, CI > 1 antagonism) (middle). Apoptosis was measured by CaspaseGlo assay, 18 h after treatment (right). (B) Distribution of CI in a diverse panel of UM and mUM cells with distinct BAP1 status. CI was determined using the HSA method (CI > 10 synergism, 0 < CI < 10 additivity, CI < 0 antagonism). (C) CI in a panel of UM cells combining darovasertib or Go6983 with various FAKi in OMM1.3, OMM1.5, and Mel202 cells determined using the HSA method. CI of PKCi combined with BRAFi (dabrafenib, vemurafenib) used as a comparison. (D) Apoptosis of UM cells measured by CaspaseGlo-3/7 assay, in response to vehicle, 1 μM VS-4718, 1 μM darovasertib, or 1 μM VS-4718 + 1 μM darovasertib for 24 h (mean ± SEM, n = 3). (E) Immunoblot showing cleaved-PARP, pFAK, and pERK in response to treatment with 1 μM VS-4718, 1 μM darovasertib, or 1 μM VS-4718 + 1 μM darovasertib for 24 h in UM cells.

Based on our emerging results, we first examined the effect of co-targeting FAK- and PKC/PKN-regulated signaling axes. We found that the combination of FAKi using VS-4718 and darovasertib synergistically inhibited cell viability and promoted apoptosis in UM cells (Figure 5A). We expanded our analysis to include a number of UM cell lines with distinct genetic BAP1 status and a recently developed syngeneic model of UM, capturing representation of all major UM genetic subtypes.^49^ We found strong synergistic profiles across all UM cell lines tested, suggesting that co-targeting FAK and PKC/PKN may be active in mUM (Figure 5B). We also observed that the combination of VS-4718 and darovasertib was able to synergistically inhibit growth under 3D growth conditions (Figures S3A and S3B). We further interrogated multiple clinically relevant FAKi versus PKCi (darovasertib and Go6983) in three UM cell lines. We observed consistent synergistic antiproliferative effects in UM cells with the combination of FAKi with darovasertib as well as Go6983, albeit the synergistic score was more significant for darovasertib (Figure 5C). However, synergism was not observed in UM cells with the combination of BRAF and PKCis as a specificity control (Figure 5C). We next tested the ability of VS-4718 and darovasertib to induce apoptotic cell death. Using a CaspaseGlo sensor for capase-3/7 cleavage as a measure of apoptosis, we observed significant induction of apoptosis aligned with our synergism results (Figure 5D). Complementing this, we assessed the ability of FAK and darovasertib to induce apoptosis measuring levels of cleaved PARP, a major substrate of caspases. In most systems, FAKi have been shown to be primarily cytostatic in nature as single agents. In line with this, we observed minimal apoptotic effects using VS-4718 as a single agent; however, the combination of darovasertib and FAKi potently induced apoptosis in several UM cell lines, including a BAP1 mutant cell line, emphasizing the potential of this combination in the context of mUM (Figure 5E). Interestingly, one cell line in our panel, OMM1.3, demonstrated greater sensitivity to single-agent darovasertib treatment. Despite this, the majority of cell lines required both FAK and darovasertib to promote apoptosis, supporting the use of the combination as a robust therapy against UM. Of interest, darovasertib is currently in clinical trials in mUM patients in combination with crizotinib with early promising results.^50^ Crizotinib is used as a MET and ALK kinase inhibitor, but an unbiased activity-based protein kinase profiling strategy showed that FAK act as a direct target of crizotinib.^51^ Thus, we asked if the combination of darovasertib and crizotinib is synergistic and related to inhibition of FAK. We found that, in UM cells, crizotinib treatment inhibited pFAK levels (Figure S3C), and that both synergy and apoptosis with the combination of crizotinib and darovasertib can only be achieved at high doses of crizotinib, at which it promotes FAK inhibition (Figures S3D−S3F). This suggests that FAK inhibition may represent a target for crizotinib to induce cell death as part of its combination with darovasertib.

### FAKi and darovasertib are synergistic in UM *in vivo* preclinical models

**Figure 6:**
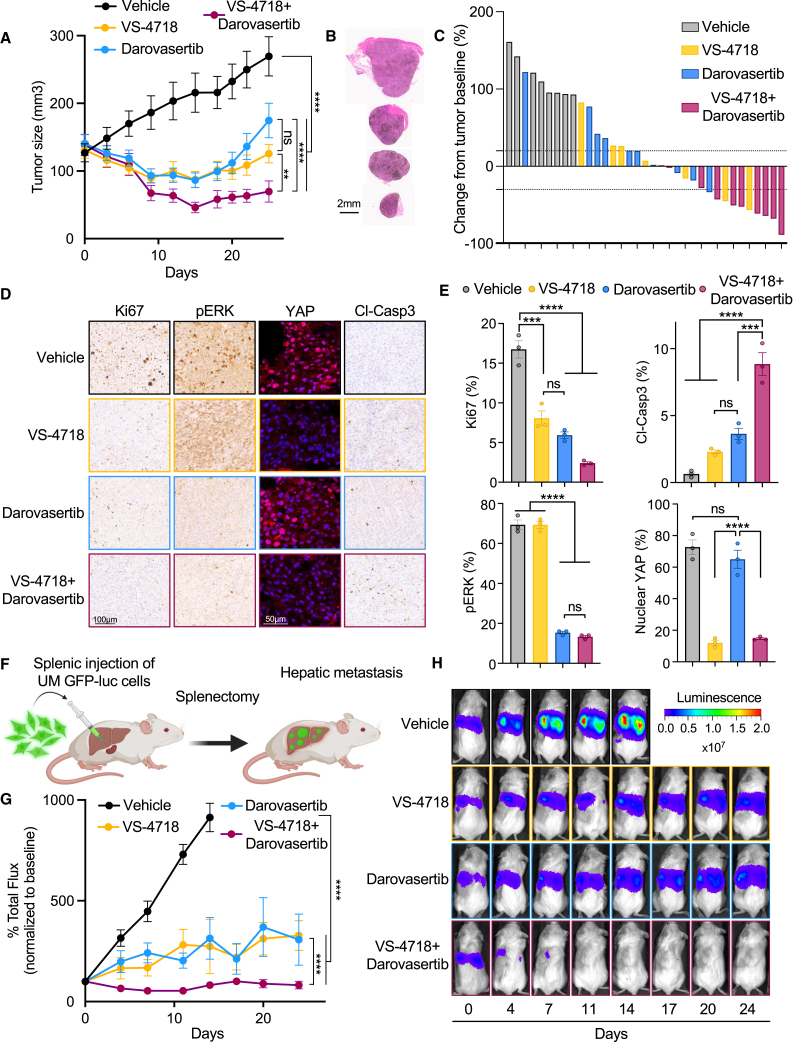
Impact of darovasertib and VS-4718 on UM tumor growth in preclinical models (A) UM 92.1 tumor xenograft growth kinetics in SCID/NOD mice treated with vehicle (control), VS-4718 50 mg/kg BID PO, darovasertib 50 mg/kg BID PO, or combination of VS-4718 50 mg/kg BID PO + darovasertib 50 mg/kg BID PO. Data are mean ± SEM (>5 mice/group). (B) H&E staining of representative xenograft tumor sections from (A) after 25 days of treatment.Scale bar, 2mm. (C) Waterfall plot depicting tumor objective response rate from (A). (D) Representative IHC staining tumor sections for Ki67, pERK, and cleaved caspase-3 (cl-Casp3). Scale bar, 100 μm. YAP was detected by immunofluorescence. Scale bar, 50 μm. (E) Quantification of stained tumor sections in (D). Control is in gray, VS-4718 is in gold, darovasertib is in blue, and VS-4718 + darovasertib combination is in magenta (mean ± SEM, n = 3). (F) Schematic of UM metastatic model. Splenic injection of 92.1 GFP-luc cells is followed by a short period of hematogenous dissemination, splenectomy, and subsequent monitoring of hepatic metastasis by IVIS. Created with Biorender. (G) Hepatic tumor burden measured by IVIS imaging after injection of 92.1 GFP-luc UM cells in SCID/NOD mice. Mice were treated with vehicle (control) VS-4718 50 mg/kg BID PO, darovasertib 50 mg/kg BID PO, or combination of VS-4718 50 mg/kg BID PO + darovasertib 50 mg/kg BID PO. Data are mean ± SEM (>5 mice/group). (H) IVIS imaging of representative mice from (G).

**Figure 7:**
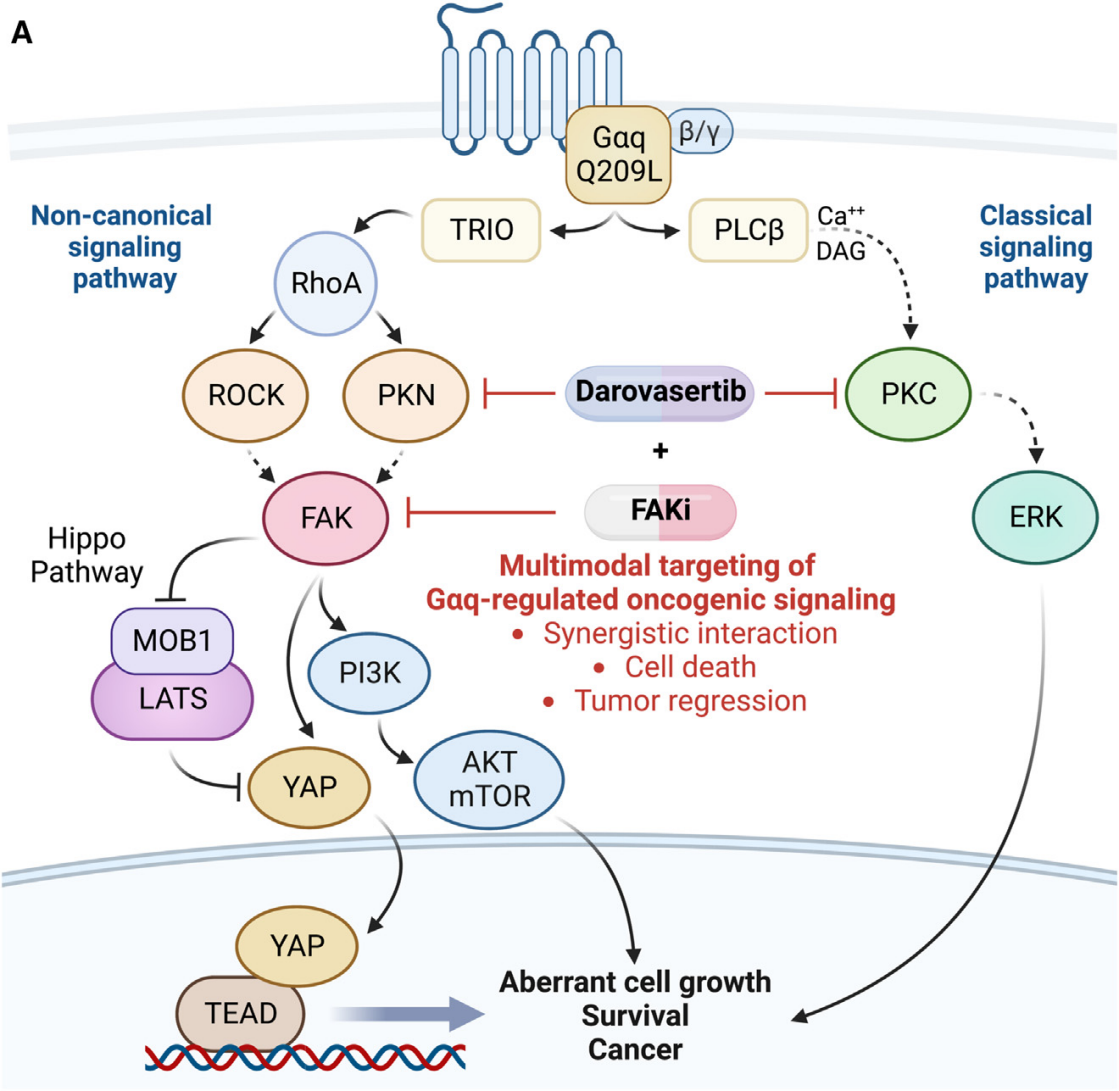
Parallel and converging signaling mechanisms driven by Gαq (A) Lateral inhibition of Gαq-regulated signaling mechanisms represents a promising signal-transduction-based precision therapy against UM. The multi-targeted kinase activity of darovasertib primes its activity on specific Gαq-regulated growth-promoting signaling networks and, in combination with FAKi, target the core survival mechanisms in UM. Created with Biorender.

To further evaluate the anticancer activity of FAKi/darovasertib, we used *in vivo* UM models reflecting clinically relevant stages of UM disease. First, we used a UM xenograft model using a human primary UM cell line 92.1. Tumor-bearing mice were randomized into four groups: vehicle control, VS-4718, darovasertib, and the VS-4718 + darovasertib combination, and administered drugs at doses reflecting usage in humans in the clinic. Over the course of treatment, while single-agent FAK and PKC/PKN inhibition was sufficient to induce partial control of tumor growth, only the combination was able to achieve and sustain tumor regression (Figures 6A–6C).^52^ No significant changes in body weight of treated mice were observed, suggesting that treatments were well-tolerated by mice with minimal adverse events (Figure S4A). To monitor the signaling pathways in the xenograft tumors, we assessed levels of key MEK/ERK, FAK/YAP, and apoptotic pathway proteins by immunohistochemistry. As anticipated, treatment groups with FAKi resulted in nuclear exclusion of YAP (Figures 6D, 6E, and S4B).^14^ Darovasertib-treated groups demonstrated a significant decrease in pERK, which is aligned with PKC-mediated control of canonical Gαq-driven signaling to MAPK (Figures 6D and 6E). Only the combination of VS-4718 + darovasertib resulted in a significant decrease in proliferating Ki67+ cells concomitant with a significant increase in cleaved caspase-3 as a marker of apoptosis (Figures 6D and 6E). We next interrogated the efficacy of the VS-4718 + darovasertib combination treatment on a mUM model. This model takes advantage of GFP-Luciferase-expressing UM cells that exhibit a highly specific hepatotropism, which is aligned with clinical presentation of mUM in humans (Figure 6F).^53^ Using this model, we observed a significant reduction in metastatic burden with the combination of VS-4718 + darovasertib (Figures 6G and 6H). Similar to the tumor response observed in our xenograft model, VS-4718 and darovasertib alone were predominantly cytostatic, whereas the combination induced potent and sustained tumor regression (Figures 6G and 6H). Finally, taking advantage of a *Gna11-*mutant syngeneic model of melanoma, we tested the combination of VS-4718 + darovasertib and observed a significant reduction in tumor burden with the combination treatment (Figures S5A−S5D).^49^ The use of a syngeneic model in immunocompetent mice enabled us to assess the impact of the combination therapy on the tumor immune microenvironment. We found no significant changes in immune cell infiltration, which supports that the usage of FAKi/darovasertib in UM patients may not interfere with newly approved immunotherapies for UM (Figures S5E−S5I). Taken together, our findings demonstrate that the combination of VS-4718 + darovasertib induces cytotoxic activity *in vitro* and *in vivo*, which cannot be achieved by administration of each single agent alone and support the future investigation evaluating the efficacy of this combination in the clinical setting (Figure 7A).

## Discussion

Although Gαq is the oncogenic driver in UM, a cancer type with limited genetic aberrancies, Gαq itself is not directly druggable with clinically ready compounds. Coupled with the prior failure of MEK inhibitors in clinical trials, the survival of mUM patients is still dismal.^20^^,^^21^ Hence, identifying key signaling hubs within its downstream pro-proliferative signaling circuits may provide a precision therapy approach for mUM and other *GNAQ*-driven malignancies.

Here, we took advantage of a clinically oriented, oncology-focused compound library and performed high-throughput single-agent and combinatorial chemogenetic drug screens to broadly interrogate the druggable landscapes of *GNAQ*-mutant UM versus *BRAF*-mutant SKCM.^31^ The unique nature of our screen facilitates the identification of oncogene-specific survival mechanisms that can be exploited as therapeutic vulnerabilities. In the context of SKCM, we identified enrichment of drug classes targeting mechanistic underpinnings of SKCM, including BRAF and MEK drug sets. We also identified targets that have not yet been extensively studied as targeted therapies in SKCM, including GSK3B and HSP90AB1, which warrant further investigation.^54^^,^^55^ Our focal effort in identifying druggable molecular mechanisms that may demonstrate synthetic lethality with oncogenic *GNAQ* revealed a number of targets that can be translated into the clinic, including epigenetic modifiers, such as HDAC- and BRD-targeting drug sets, which are being recently explored, as well as significant enrichment of PKC and MCL1 inhibitors.^56^^,^^57^^,^^58^^,^^59^^,^^60^^,^^61^ A number of these target sets were represented among the top UM-targeting drugs, including S63845, a preclinical MCL1 inhibitor, and BMS-986158 and AZD-5153, both BRD4-targeting drugs in clinical trials.^62^^,^^63^ Guided by our finding that darovasertib, a PKCi, demonstrated superior performance compared with all other agents in our screen, and the proximity of PKC as a signal transducer of Gαq signaling, we focused on this agent as a candidate precision therapy in UM.

Our investigation interrogating the enhanced activity of darovasertib compared with all other PKCi in UM revealed that it acts as a dual inhibitor of both PKC and PKN, the latter a closely related group of kinases in the AGC family of serine/threonine kinases. Compared to PKCs, PKNs are relatively poorly investigated, and PKN family members are considered part of the “dark kinome,” thus presenting an exciting opportunity to expand our understanding of their role in the context of larger signaling networks.^64^ We find here that, in both UM and HEK293 cells, the latter as a model system, PKNs are activated downstream of Gαq/RhoA. In addition, although there has been a recently proposed role of PKC regulating FAK in UM,^65^ we show here that blockade of PKN, but not PKC, decreases FAK activity, and that in turn PKN can further activate FAK. The latter is aligned with site-recognition screens of PKN family kinases, which revealed FAK as a candidate PKN substrate, thus suggesting that PKN may directly regulate FAK activity in UM.^66^ Taken together, these findings support that RhoA acts as a central signaling node for non-canonical Gαq signaling, coordinating an axis that bifurcates into ROCK- and PKN-mediated control of FAK activity. This is aligned with the recent atlas of the substrate specificity of the human serine/threonine kinome, which revealed that FAK is among the top predicted substrates for PKN family kinases.^67^ Together, this establishes PKN as a novel element of the multicomponent signaling network driven by Gαq. Moreover, our finding that darovasertib concomitantly inhibits both canonical and non-canonical Gαq-driven signaling pathways (e.g., PKC and PKN/FAK, respectively), defines the mechanism underlying the unique potency of darovasertib compared with other PKCi in UM. Indeed, while previous PKCi tested in UM patients have failed to demonstrate significant clinical responses, our observations revealing the unique activity profile of darovasertib may explain its more promising early clinical activity in ongoing trials.^35^^,^^68^

While the blockade of PKC/PKN by darovasertib disrupts Gαq-driven signaling axes, we find that darovasertib alone is insufficient to promote a sustained inhibition of YAP activity downstream of FAK. Effective blockade of these signaling axes is critical to abrogate potential mechanisms of resistance mediated by downstream effectors of Gαq-FAK, including the PI3K/AKT pathway.^23^ This is aligned with recent work demonstrating that PKCi monotherapy is not sufficient to suppress the multiple Gαq-regulated growth-promoting pathways in UM.^69^ Instead, the partial reduction of FAK activation by darovasertib may sensitize FAK for its further inhibition by direct ATP-competitive kinase inhibitors, thus reinforcing a multipronged pharmacology paradigm that ultimately requires co-targeting both Gαq-regulated signaling axes to achieve a durable clinical benefit.^53^ This possibility is supported by the potent synergism we observe by co-targeting PKC/PKN- and FAK-mediated signaling pathways *in vitro* and *in vivo* xenograft and mUM models resulting in UM cell death and tumor regression.

Clinical trials using darovasertib as a single agent and in combination with crizotinib, (NCT03947385),^34^ and FAKi as a single agent (NCT04109456), and in combination with MEK inhibitors (NCT04720417)^70^ in mUM patients are currently ongoing. Moreover, darovasertib was recently granted orphan-drug designation as neoadjuvant treatment of UM and mUM prior to primary interventional treatment, and fast-track designation for darovasertib in combination with crizotinib for the treatment of mUM, which may support the clinical translatability of our findings.^71^^,^^72^ While these trials are being conducted, there remains an urgent need to identify additional treatment options for mUM patients. Our current study supports the rationale of clinically evaluating the combination of darovasertib with FAKi as a potentially powerful signal transduction-based combination therapy to control unresectable primary UM and mUM patients. The combination of darovasertib and FAKi was recently found to have efficacy in complementary UM PDX models, thus further supporting the clinical potential of the combination strategy.^73^ In addition, our studies in syngeneic mouse melanoma models driven by mutant *Gna11* support that the darovasertib/FAKi combination does not affect the tumor immune landscape, which raises the possibility of using this combination in patients that progress while on treatment with recently approved immunotherapies.^49^ In addition, our pharmacogenomics screen and mechanistic analysis raises the benefits of targeting aberrant ERK signaling via PKC instead of prior MEK-targeting agents tested in UM patients. Indeed, this may represent an attractive option due to the proximity of PKC as a major downstream signal transducer of mutant Gαq signaling to ERK, rather than suppressing ERK function through MEK inhibition systemically, thus expanding the clinical spectrum of patients likely to benefit from this combination with lower toxicities.^74^ We also obtained evidence that the enhanced cytotoxic activity of the combination of darovasertib with crizotinib with respect to darovasertib as single agent can be explained, at least in part, by the ability of crizotinib to inhibit FAK. Thus, the possibility exists that darovasertib combined with FAKi may be similarly or even more efficacious than when combined with crizotinib, but with lower toxicities. Overall, these exciting possibilities warrant further investigation.

Within this framework, our findings, in the context of emerging studies investigating the complex signaling circuits underlying Gαq-driven UM, substantiate the usage of rational combination therapies to control primary UM and mUM. Indeed, as the field of precision medicine evolves to encompass the numerous molecular drivers of cancer, this will be key to achieving durable clinical responses. Taken together, our findings also demonstrate the utility of chemogenetic drug screens and network-based approaches to identify pharmacological vulnerabilities, thereby strengthening the clinical toolbox against primary and mUM and working toward filling a large therapeutic gap for this *GNAQ*-driven malignancy.

### Limitations of the study

Potential limitations of our study include the usage of xenograft and syngeneic mouse models of UM, as they do not fully recapitulate the characteristics of human UM tumors. Investigation of the combination of darovasertib with FAK in patient-derived xenografts, genetically engineered mouse models, or other clinically relevant models would increase the translatability to human pathophysiology. Moreover, while we demonstrate that the potency of darovasertib in UM is driven by its multi-targeted activity against PKC and PKN, it is possible that darovasertib has additional targets not included in our kinome analysis contributing to this effect. Further investigation may provide additional insights. Taken together, while our study has some limitations, it establishes a strong foundation for the investigation of darovasertib + FAKi combination as a promising therapeutic strategy for UM patients.

## STAR★Methods

### Key resources table

**Table:** 

REAGENT or RESOURCE	SOURCE	IDENTIFIER
**Antibodies**		

FAK	Cell Signaling Technology	Cat#: 71433, RRID:AB_2799801
pY397-FAK	Cell Signaling Technology	Cat#: 8556, RRID: AB_10891442
ERK1/2	Cell Signaling Technology	Cat#: 9102, RRID:AB_330744
pT202/Y204-ERK1/2	Cell Signaling Technology	Cat#: 4370, RRID:AB_2315112
GAPDH	Cell Signaling Technology	Cat#: 5174, RRID:AB_10622025
PKN2	Cell Signaling Technology	Cat#: 2612, RRID:AB_2167753
PKCε	Cell Signaling Technology	Cat#: 2683, RRID:AB_2171906
PKCδ	Cell Signaling Technology	Cat#: 9616, RRID:AB_10949973
MEK1/2	Cell Signaling Technology	Cat#: 9126, RRID:AB_331778
pS217/221-MEK1/2	Cell Signaling Technology	Cat#: 9154, RRID:AB_2138017
Cl-PARP	Cell Signaling Technology	Cat#: 5625, RRID:AB_10699459
YAP	Cell Signaling Technology	Cat#: 14074, RRID:AB_2650491
pS127-YAP	Cell Signaling Technology	Cat#: 13008, RRID:AB_2650553
BAP1	Cell Signaling Technology	Cat#: 13271, RRID:AB_2798168
β-actin	Cell Signaling Technology	Cat#: 4970, RRID:AB_2223172
Vinculin	Cell Signaling Technology	Cat#: 13901, RRID:AB_2728768
PKN1	ThermoFisher Scientiifc	Cat#: MA5-19703, RRID:AB_2607709
pPKN1/2/3	Abcam	Cat#: ab187660
PKN3	Novus Biologicals	Cat#: NBP1-30102, RRID:AB_2163985
HRP-conjugated goat anti-rabbit IgG	Southern Biotech	Cat#: 4010-05, RRID:AB_2632593
HRP-conjugated goat anti-mouse IgG	Southern Biotech	Cat#: 1010-05, RRID:AB_2728714
Ki67	Dako Technologies	Cat#: M724029-2
Cleaved Caspase-3	Cell Signaling Technology	Cat#: 9661, RRID:AB_2341188
Goat anti-Rabbit IgG (H + L) Highly Cross-Adsorbed Secondary Antibody, Alexa Fluor™ Plus 488	ThermoFisher Scientific	Cat#: A32731, RRID:AB_2633280
CD45-Alexa Fluor 700	Biolegend	clone 30-F11, Cat#: 103127, RRID:AB_493714
Thy1.2-PerCP-Cy5.5	Biolegend	clone 30-H12, Cat#: 105337, RRID:AB_2571944
CD19-BV510	Biolegend	clone 6D5, Cat#: 115545, RRID:AB_2562136
CD4-APC-Fire 750	Biolegend	clone RM4-4, Cat#: 116019, RRID:AB_2715955
CD8a-BUV737	BD Biosciences	clone 53-6.7, Cat#: AB_2870090
CD11b-BV711	Biolegend	clone M1/70, Cat#: 101241, RRID:AB_11218791

**Chemicals, Peptides, and Recombinant Proteins**		

Darovasertib (LXS-196)	Selleck Chemicals	Cat#: S6723
Go6983	Selleck Chemicals	Cat#: S2911
Sotrastaurin	Selleck Chemicals	Cat#: S2791
ROCKi (Y-27632)	Selleck Chemicals	Cat#: S6390
VS-4718	Verastem Oncology	Gift
Defactinib	Verastem Oncology	Gift
C3 toxin	Cytoskeleton Inc	Cat#: CT04-A
FR900359	Dr. Evi Kostenis	Gift
Hoechst 33342	ThermoFisher Scientific	Cat#: H1399
basic fibroblast growth factor	ThermoFisher Scientific	Cat#: 13256029
epithelial growth factor	ThermoFisher Scientific	Cat#: PHG0313
B-27	ThermoFisher Scientific	Cat#: 17504044
N2 Supplement	ThermoFisher Scientific	Cat#: 17502-048
D-luciferin potassium salt	GoldBio	Cat#: LUCK-100

**Critical Commercial Assays**		

CaspaseGlo3/7 assay system	Promega	Cat#: G8090
AquaBluer Cell Viability Reagent	Boca Scientific Inc.	Cat#: 6015

**Deposited Data**		

Mass spectrometry data	This paper	PRIDE: PXD038023

**Experimental Models: Cell Lines**		

HEK293	SigmaAldrich	Cat#: 85120602
A375	NIH Cell Bank	N/A
WM266	NIH Cell Bank	N/A
SKMEL28	NIH Cell Bank	N/A
92.1	Laboratory of Dr. Bruce Ksander	Vaque et al.^22^
OMM1.3	Laboratory of Dr. Bruce Ksander	Vaque et al.^22^
OMM1.5	Laboratory of Dr. Bruce Ksander	Vaque et al.^22^
Mel202	Laboratory of Dr. Bruce Ksander	Vaque et al.^22^
Hmel1274/M3	Laboratory of Dr. Glenn Merlino	Perez-Guijarro et al.^49^
MP46	ATCC	Cat#: CRL-3298
MP38	ATCC	Cat#: CRL-3296
MM28	ATCC	Cat#: CRL-3295
MP41	ATCC	Cat#: CRL-3297

**Experimental Models: Organisms/Strains**		

Mouse: NOD.Cg-*Prkdc*^*scid*^*Il2rg*^*tm1Wjl*^/SzJ	UCSD In-house breeding program	N/A
Mouse: C57BL/6	UCSD In-house breeding program	N/A

**Oligonucleotides**		

siRNA: Non-targeting Control	Horizon Discovery Biosciences	Cat#: D-001810-10-05
siRNA: PRKCD	Horizon Discovery Biosciences	Cat#: L-003524-00-0005
siRNA: PRKCE	Horizon Discovery Biosciences	Cat#: L-004653-00-0005
siRNA: PKN1	Horizon Discovery Biosciences	Cat#: L-004175-00-0005
siRNA: PKN2	Horizon Discovery Biosciences	Cat#: L-004612-00-0005
siRNA: PKN3	Horizon Discovery Biosciences	Cat: L-004647-00-0005

**Recombinant DNA**		

pCEFL-EV	Feng et al.^15^	N/A
pCEFL-GαqQL	Feng et al.^15^	N/A
pCEFL-RhoA-QL	Marinissen et al.^44^	N/A
pCEFL-myr-PKN2-FL	Marinissen et al.^44^	N/A
pLVX-RhoA-FLAG	This paper	N/A

### Resource availability

#### Lead contact

Further information and requests for resources and reagents should be directed to and will be fulfilled by the lead contact, J. Silvio Gutkind (sgutkind@health.ucsd.edu).

#### Materials availability

Plasmids generated in this study will be available on request through completion of a Material Transfer Agreement.

### Experimental model and study participant details

#### Cell lines, culture Procedures and chemicals

HEK293 (female), A375 (female), WM266 (female) and SKMEL28 (male) cells were cultured in DMEM (D6429, Sigma-Aldrich Inc.) containing 10% FBS (F2442, Sigma-Aldrich Inc.), 1X antibiotic/antimycotic solution (A5955, Sigma-Aldrich Inc.), and 1X Plasmocin prophylactic (ant-mpp, InvivoGen). Uveal melanoma cells (92.1 (female), OMM1.3 (male), OMM1.5 (male), and Mel202 (female)) and mouse melanoma cells (Hmel1274/M3)^49^ were cultured in RPMI-1640 (R8758, Sigma Aldrich Inc.) containing 10% FBS (F2442, Sigma-Aldrich Inc.), 1X antibiotic/antimycotic solution (A5955, Sigma-Aldrich Inc.), and 1X Plasmocin prophylactic (ant-mpp, InvivoGen). MP46 (male), MP38 (male), MM28 (male), and MP41 (female) were cultured in RPMI-1640 (R8758, Sigma Aldrich Inc.) containing 20% FBS (F2442, Sigma-Aldrich Inc.), 1X antibiotic/antimycotic solution (A5955, Sigma-Aldrich Inc.), and 1X Plasmocin prophylactic (ant-mpp, InvivoGen). All cell lines were routinely tested free of mycoplasma contamination. Darovasertib (LXS-196) (S6723), Go6983 (S2911), Sotrastaurin (S2791) and ROCKi (Y-27632) (S6390) were purchased from SelleckChem. VS-4718 and defactinib were provided by Verastem Oncology. C3 toxin (CT04-A) was purchased from Cytoskeleton Inc. FR900359 was a kind gift from Dr. Evi Kostenis.

#### Human xenograft tumor models

All animal studies were approved by the Institutional Animal Care and Use Committee of University of California, San Diego (San Diego, CA) with protocol S15195 and were performed in accordance with relevant guidelines and regulations. Female 4- to 6-week-old NOD.Cg-Prkdcscid Il2rgtm1Wjl/SzJ (SCID-NOD) mice were purchased from the UCSD in-house breeding program. Mice were injected subcutaneously in both flanks with 1 × 10^6^ 92.1 cells. For syngeneic tumor allograft studies, C57BL/6 were purchased from the UCSD in-house breeding program, and mice were injected subcutaneously in both flanks with 0.75 × 10^6^ with Hmel1274/M3 cells. Mice were monitored 2–3 times per week for tumor development. Tumor growth analysis was assessed as LW^2^/2, where L and W represent length and width of the tumor. VS-4718 and darovasertib were prepared in 0.5% CMC (Sigma-Aldrich) and 0.1% Tween 80 (Sigma-Aldrich) in sterile water. Mice were administered 50 mg/kg VS-4718 (Verastem Oncology) and/or 50 mg/kg darovasertib twice daily by oral gavage; control group was treated with vehicle. Mice were euthanized at the indicated time points and tumors were isolated for histologic, and IHC evaluation. Results of mice experiments were expressed as mean ± SEM of a total of tumors analyzed.

#### Metastatic UM model

Female 4- to 6-week-old SCID-NOD mice were injected with 1 × 10^6^ 92.1 GFP-Luc cells in the spleen, followed by splenectomy at 2 min post injection. Tumor implantation by bioluminescence was assessed twice weekly by bioluminescence images captured using the *In Vivo* Imaging System (IVIS) (PerkinElmer). To this end, mice received an intraperitoneal injection of 200 mg/kg D-luciferin potassium salt diluted in sterile PBS 15 min before imaging (GoldBio, LUCK-100). On day 10 post-surgery, baseline bioluminescence was measured, and mice were randomized. Mice were excluded from the study if no signal was detectable on day 10 post-surgery. Treatments were administered, with the above-mentioned dosing.

### Method details

#### Plasmids and Transfections

Plasmids pCEFL-EV, pCEFL-GαqQL, pCEFL-RhoA-QL, and pCEFL-myr-PKN2-FL were described previously.^15^^,^^22^^,^^44^ pLVX-RhoA-FLAG was generated using the Gateway Cloning system (Life Technologies) to subclone human RhoA into a doxycycline-inducible N-terminal 3XFLAG-tagged vector modified to be Gateway compatible from the pLVX-puro vector (Clontech). For overexpression experiments, HEK293 cells were transfected with Turbofect (R0531, Thermofisher Scientific, CA) according to manufacturer instructions. All knockdown experiments were performed using siRNAs purchased from Horizon Discovery Biosciences (Non-targeting Control: D-001810-10-05, PRKCD: L-003524-00-0005, PRKCE: L-004653-00-0005, PKN1: L-004175-00-0005, PKN2: L-004612-00-0005, PKN3: L-004647-00-0005), and Lipofectamine RNAiMAX Reagent (13778150, Thermofisher Scientific, CA) according to manufacturer’s instructions.

#### Quantitative high-throughput single-agent screening (qHTS) and drug combination studies

For single-agent qHTS, cells were seeded in 5 μL of growth media using a Multidrop Combi dispenser (ThermoFisher) into 1536-well white polystyrene tissue culture-treated plates (Corning) at a density of 500-cells/well. Cells were then incubated overnight in a 5% CO2 incubator to enable reattachment to the plate. The following day, 23 nL of MIPE 5.0 compounds were added to individual wells (11 doses tested for each compound in separate wells, for a total of 22x1536-well plates for each cell line screened) via a 1536 pin-tool. Bortezomib (final concentration 2.3 μM) was used as a positive control for cell cytotoxicity. Plates were incubated for 48 h at standard incubator conditions covered by a stainless steel gasketed lid to prevent evaporation. 48h post compound addition, 3 μL of Cell Titer Glo (Promega) were added to each well and plates were incubated at room temperature for 15 min with the stainless-steel lid in place. Luminescence readings were taken using a Viewlux imager (PerkinElmer) with a 2 s exposure time per plate. Relative viability was assessed by CellTiterGlo (Promega) with respect to DMSO treated wells (column #4 in each plate). Cell context-specific hits were extracted based on a cutoff of ΔAUC>±40 and filtered based on curve classification criteria of −1.1, −1.2, −2.1 and −2.2 for at least 2 cell lines in either cell context.^77^

For drug combination screening, 10 nL of compounds were acoustically dispensed into 1536-well white polystyrene tissue culture-treated plates with an Echo 550 acoustic liquid handler (Labcyte). Cells were then added to compound-containing plates at a density of 500-cells/well in 5 μL of medium. A 10-point custom concentration range, with constant 1:2 dilution was used for all the drug combination pairs assessed in 10 × 10 matrix format. Synergistic cytotoxicity was assessed by Cell Titer Glo at 48h post drug treatment, exactly as described above for single agent qHTS.

For apoptosis induction assessments, 3 μL of Caspase3/7 Glo (Promega) were added to each well 18h post-treatment and plates were incubated at room temperature for 15 min with the stainless-steel lid in place. Luminescence readings were taken using a Viewlux imager (PerkinElmer) with a 10 s exposure time per plate.

#### Secondary viability assessments

For selected agents, cells were seeded at a density of 5 × 10^3^ to 1 × 10^4^ cells/well in 96-well white plates. Eight different dilutions of each inhibitor were assayed in technical triplicates for 72 h in each experiment. Cell viability was measured with the AquaBluer Cell Viability Reagent on a Spark microplate reader (Tecan). Using the GraphPad Prism v8.2.0 software, the half-maximal inhibitor concentration values (GI _50_) were determined from the curve using the nonlinear log (inhibitor) versus response–variable slope (three parameters) equation. GI _50_ values were only determined for compounds that inhibited growth by more than 50%.

#### Identification of target-level dependencies

To enable the unbiased identification of target-level dependencies in UM and SKCM cell-lines, we purposely exploited MIPE 5.0 mechanistic redundancy to create a custom collection of drug-target sets, representing any MIPE 5.0 drug-target that is covered by at least 3 small-molecule drugs (n = 278). We then ranked the entire MIPE 5.0 outcomes based on the differential Z-AUC score between UM and SKCM cell lines (difference between the Z-AUC average of each group). We used this ranked list to run a pre-ranked, GSEA-like enrichment analysis against the custom collection of drug-target sets described above. The pre-ranked enrichment analysis was performed using the GSEA software (v4.0.3) with a weighted enrichment statistic.

#### Protein interaction studies

Affinity purification and downstream mass spectrometry analysis was performed as previously described.^78^ Cells lysed with 500 μL of ice-cold lysis buffer (50 mM Tris pH 7.4, 150 mM NaCl, 1 mM EDTA, 0.5% NP40, 1x protease inhibitor cocktail (Roche, complete mini EDTA free), 125U Benzonase/mL). Lysates were flash-frozen twice on dry ice for 5–10 min, followed by a 30–45 s thaw in 37°C water bath with agitation. Lysate was clarified by centrifugation at 13000 x g for 15 min at 4°C.

For FLAG purification, 30 μL of bead slurry (Anti-Flag M2 Magnetic Beads, Sigma) was washed twice with 1 mL of ice-cold wash buffer (50 mM Tris pH 7.4, 150 mM NaCl, 1 mM EDTA) and the lysate was incubated with the anti-FLAG beads at 4°C with agitation for 2 h on a KingFisher Flex. After incubation, flow-through was removed and beads were washed once with 1 mL of wash buffer with 0.05% NP40 and twice with 1 mL of wash buffer (no NP40). Bound proteins were eluted by incubating beads with 30 μL of 100 μg/ml 3xFLAG peptide in 0.05% RapiGest in wash buffer for 15 min at RT with shaking. Supernatants were removed and elution was repeated with 15 μL. Eluates were combined and 15 μL of 8 M urea, 250 mM Tris, 5 mM DTT (final concentration ∼1.7 M urea, 50 mM Tris, and 1 mM DTT) was added to give a final total volume of 50 μL. Samples were incubated at 60°C for 15 min and allowed to cool to room temperature. Iodoacetamide was added to a final concentration of 3 mM and incubated at room temperature for 45 min in the dark. DTT was added to a final concentration of 3 mM before adding 1 μg of sequencing-grade trypsin (Promega) and incubating at 37°C overnight. Samples were acidified to 0.5% TFA (ph < 2) with 10% TFA stock and incubated for 30 min before desalting on C18 stage tip (Rainin).

Samples were then resuspended in 20 μL of MS loading buffer (4% formic acid, 2% acetonitrile) and 2μL were separated by a reversed-phase gradient over a nanoflow 75μm ID x 25cm long picotip column packed with 1.9μM C18 particles (Dr. Maisch). Peptides were directly injected over the course of a 70 min acquisition into an Orbitrap Fusion Tribrid mass spectrometer (Thermo). Raw MS data were searched against the uniprot canonical isoforms of the human proteome (downloaded March 21, 2018) using the default settings in MaxQuant^79^ (version 1.6.6.0). Peptides and proteins were filtered to 1% false discovery rate in MaxQuant, and identified proteins were then subjected to protein-protein interaction scoring. Protein spectral counts as determined by MaxQuant search results were used for PPI confidence scoring by SAINTexpress^80^(version 3.6.1), using samples in which RhoA expression was not induced by addition of doxycycline as controls. The list of PPIs was filtered to those with a SAINTexpress BFDR =< 0.05. All raw mass spectrometry proteomics data files have been deposited to the ProteomeXchange Consortium via the PRIDE partner repository with the dataset identifier PXD038023.^81^^,^^82^

#### Kinome profiling

The principal method utilized for kinome profiling is a radioactive filter binding assay using 33P ATP, described previously.^38^^,^^39^ Protein kinase profiling of LXS-196 was undertaken at a concentration of 1 μM and carried out against the Dundee panel of 140 protein kinases at the International Center for Protein Kinase Profiling (www.kinase-screen.mrc.ac.uk/). Results for each kinase are presented as the *Z* score of mean kinase activity ±S.D. for an assay undertaken in duplicate relative to a control kinase assay in which the inhibitor was omitted. Abbreviations and assay conditions used for each kinase are available (http://www.kinase-screen.mrc.ac.uk/services/premier-screen).

#### 3D growth assay

Cells were seeded in 96-well ultra-low attachment plate (#CLS3474, Corning, Tewksbury, MA) at 50 cells/well with sphere medium consisted of DMEM/F12 Glutamax (#10565042, Thermo Fisher Scientific), 20 ng/mL basic fibroblast growth factor (#13256029, Thermo Fisher Scientific), 20 ng/mL epithelial growth factor (#PHG0313, Thermo Fisher Scientific), B-27 (#17504044, Thermo Fisher Scientific), and N2 Supplement (#17502-048, Thermo Fisher Scientific). Drug was added the day after cells were seeded. After 20 days, images were acquired, and size of spheres were quantified using ImageJ.

#### Immunoblotting and Immunoprecipitations

Cells were serum starved, and then treated according to the conditions in the figure legend. For cell lysis, cells were washed 2X in cold PBS and lysed in 1Xcell Lysis buffer (Cell Signaling Technologies, 9803) supplemented with HaltTM Protease and Phosphatase Inhibitor Cocktail (#78440, ThermoFisher Scientific) and 1mM Sodium Orthovanadate (P0758S, New England Biolabs). Lysates were centrifuged at max speed at 4°C, concentrations were measured using DC Protein Assay (BioRad Laboratories, 5000111) and lysates were prepared with addition of 4x Laemmli Sample Buffer (#1610747, BioRad Laboratories), and boiled for 5 min at 98°C.

For immunoblotting, cell lysates were subjected to SDS/PAGE on 10% acrylamide gels and electroblotted to PVDF membranes. Blocking and primary and secondary antibody incubations of immunoblots were performed in Tris-buffered saline +0.1% Tween 20 supplemented with 5% (w/v) BSA or 5% w/v skim milk. The following primary antibodies were all purchased from Cell Signaling Technologies and used at 1:1000. FAK (71433), pY397-FAK (8556), ERK1/2 (9102), pT202/Y204-ERK1/2 (4370), GAPDH (5174), PKN2 (2612), PKCε (2683), PKCδ (9616), MEK1/2 (9126), pS217/221 MEK1/2 (9154), Cl-PARP (5625), YAP (14074), pS127 YAP (13008), BAP1 (13271), Beta-actin (4970), and Vinculin (13901). PKN1 (MA5-19703) was purchased from ThermoFisher Scientific. pPKN1/2/3 (ab187660) was purchased from Abcam. PKN3 (NBP1-30102) was purchased from Novus Biologicals. HRP-conjugated goat anti-rabbit (4010-05) and anti-mouse (1010-05) IgGs (Southern Biotech, AL) were used at a dilution of 1:30,000, and immunoreactive bands were detected using Immobilon Western Chemiluminescent HRP substrate (Millipore, MA) according to the manufacturer’s instructions. All western blots were performed in at least 3 independent experiments, representative images are shown.

#### CaspaseGlo3/7 assay

Cells were seeded at a density of 10000 cells/well in 96-well white plates. After 24 h, drug treatment or vehicle was added and cells were assayed as indicated. Apoptosis was measured using the Promega CaspaseGlo3/7 Assay System (G8090) as per manufacturer’s instructions.

#### Immunohistochemistry and immunofluorescence

For immunohistochemical analysis, tumor xenografts were harvested and fixed in 10% aqueous buffered zinc formalin and paraffin embedded. Tissue processing and staining was performed as described previously using the following antibodies: Ki67 (Dako Technologies: M724029-2), pERK (Cell Signaling Technologies: 4370), Cleaved Caspase-3 (Cell Signaling Technologies: 9661).^83^^,^^84^ Samples were mounted in prolong gold anti-fade mounting medium (Invitrogen) and were scanned using a Zeiss Axioscan Z1 slide scanner equipped with a 20×/0.8 NA objective. For YAP staining from tumor xenografts, tissues were embedded in OCT and flash frozen, and stained using YAP antibody (Cell Signaling Technology, 14074) as previously described.^84^

For *in vitro* YAP staining, OMM1.3 uveal melanoma cells were cultured on coverslips and treated with 1μM VS-4718 or 1μM LXS-196 treatment for 24hrs, vehicle treatment was used as a control. Cells were washed with PBS, fixed with 3.7% formaldehyde in phosphate-buffered saline (PBS) for 30 min, and permeabilized using 0.05% Triton X-100 for 10 min. Fixed cells were blocked with 3% FBS-containing PBS for 30 min, and incubated with YAP antibody (Cell signaling technology, 14074), overnight. YAP was visualized with AlexaFluor488-labeled secondary antibody (ThermoFisher, A32731). Samples were mounted in PBS buffer containing Hoechst 33342 (ThermoFisher, H1399) for nuclear staining. Confocal images were acquired using an Olympus FV1000 with 405, 488, 555, and 647 laser lines. Images were linearly analyzed and pseudo-coloured using ImageJ analysis software.^85^ In all cases for quantification, at least three regions of interest (ROIs) were selected for each condition and the percentage of positive cells for the corresponding marker was calculated.

#### Immunophenotyping of tumor infiltrating leukocytes by flow cytometry

The flanks of C57Bl/6Crl mice were implanted subcutaneously with 0.75 × 10^6^ Hmel1274/M3 cells. When tumors reached an average volume of 100mm^3^, mice were randomized and treatment with VS-4718 and darovasertib was initiated. Mice were humanely euthanized by IACUC approved methods and M3 tumors were isolated after 12 days of treatment. Cell suspensions were generated by subjecting minced tumor tissue preparations to mechanical and enzymatic dissociation using the Tumor Dissociation Kit enzyme cocktail and murine tumor dissociation protocol on the gentleMACS Octo Dissociator per manufacturer recommendations (Miltenyi Biotec). Dissociated tissues were passed through 70-μm and 40-μm cell strainers to produce single-cell suspensions. Samples were washed with PBS and stained for viable cells using LIVE/DEAD Blue fixable viability dye (eBioscience). Surface staining was then performed at the indicated antibody dilutions for 30 min at 4°C with the following antibodies. CD45-Alexa Fluor 700 at 1:100 (clone 30-F11, Biolegend), Thy1.2-PerCP-Cy5.5 at 1:200 (clone 30-H12, Biolegend), CD19-BV510 at 1:100 (clone 6D5, Biolegend), CD4-APC-Fire 750 at 1:200 (clone RM4-4, Biolegend), CD8a-BUV737 at 1:100 (clone 53-6.7, BD Biosciences), CD11b-BV711 at 1:200 (clone M1/70, Biolegend). Stained cells were fixed using the Foxp3/Transcription Factor Staining Buffer Set per manufacturer recommendations (eBioscience). Flow cytometry was performed on the BD LSR Fortessa X-20. Downstream analysis was performed using FlowJo, version 10.9.0. Viable tumor infiltrating leukocytes were defined by forward and side scatter parameters and surface expression of CD45. T cells were further defined by surface expression of Thy1.2 and absence of CD19. T cell subsets were classified by CD4 or CD8 expression. B cells were defined by surface expression of CD19 and absence of Thy1.2. Myeloid cells were defined as CD11b^+^ leukocytes negative for surface expression of Thy1.2 and CD19.

#### Statistical analysis and reproducibility

All data analysis was performed using GraphPad Prism version 9.4.0 for Mac (GraphPad Software, San Diego, CA). The data were analyzed by ordinary one-way ANOVA test or t-test as appropriate. Data is represented as mean SEM unless otherwise noted. Statistical significance is indicated as: ∗ p < 0.05, ∗∗ p < 0.01, ∗∗∗ p < 0.001, ∗∗∗∗ p < 0.0001).

## Data Availability

Source data are provided with this paper. The mass spectrometry proteomics data have been deposited to the ProteomeXchange Consortium via the PRIDE^76^ partner repository publicly available as of the date of publication. Accession numbers are listed in the key resources table. This paper does not report original code. Any additional information required to reanalyze the data reported in this paper is available from the lead contact upon request.

## Supplemental information

Document S1. Figures S1−S5 and Tables S2 and S3

Table S1. UM- and SKCM-selective agents, and corresponding Z-AUC scores, related to Figure 1

Document S2. Article plus supplemental information

## References

[1] Druker B.J., Tamura S., Buchdunger E., Ohno S., Segal G.M., Fanning S., Zimmermann J., Lydon N.B. (1996). Effects of a selective inhibitor of the Abl tyrosine kinase on the growth of Bcr-Abl positive cells. Nat. Med..

[2] Haber D.A., Bell D.W., Sordella R., Kwak E.L., Godin-Heymann N., Sharma S.V., Lynch T.J., Settleman J. (2005). Molecular targeted therapy of lung cancer: EGFR mutations and response to EGFR inhibitors. Cold Spring Harb. Symp. Quant. Biol..

[3] Robertson A.G., Shih J., Yau C., Gibb E.A., Oba J., Mungall K.L., Hess J.M., Uzunangelov V., Walter V., Danilova L. (2017). Integrative Analysis Identifies Four Molecular and Clinical Subsets in Uveal Melanoma. Cancer Cell.

[4] Van Raamsdonk C.D., Griewank K.G., Crosby M.B., Garrido M.C., Vemula S., Wiesner T., Obenauf A.C., Wackernagel W., Green G., Bouvier N. (2010). Mutations in GNA11 in uveal melanoma. N. Engl. J. Med..

[5] Van Raamsdonk C.D., Bezrookove V., Green G., Bauer J., Gaugler L., O’Brien J.M., Simpson E.M., Barsh G.S., Bastian B.C. (2009). Frequent somatic mutations of GNAQ in uveal melanoma and blue nevi. Nature.

[6] Moore A.R., Ceraudo E., Sher J.J., Guan Y., Shoushtari A.N., Chang M.T., Zhang J.Q., Walczak E.G., Kazmi M.A., Taylor B.S. (2016). Recurrent activating mutations of G-protein-coupled receptor CYSLTR2 in uveal melanoma. Nat. Genet..

[7] Harbour J.W., Onken M.D., Roberson E.D.O., Duan S., Cao L., Worley L.A., Council M.L., Matatall K.A., Helms C., Bowcock A.M. (2010). Frequent mutation of BAP1 in metastasizing uveal melanomas. Science.

[8] Rantala E.S., Hernberg M., Kivelä T.T. (2019). Overall survival after treatment for metastatic uveal melanoma: a systematic review and meta-analysis. Melanoma Res..

[9] Khoja L., Atenafu E.G., Suciu S., Leyvraz S., Sato T., Marshall E., Keilholz U., Zimmer L., Patel S.P., Piperno-Neumann S. (2019). Meta-analysis in metastatic uveal melanoma to determine progression free and overall survival benchmarks: an international rare cancers initiative (IRCI) ocular melanoma study. Ann. Oncol..

[10] Carvajal R.D., Schwartz G.K., Tezel T., Marr B., Francis J.H., Nathan P.D. (2017). Metastatic disease from uveal melanoma: treatment options and future prospects. Br. J. Ophthalmol..

[11] Arang N., Gutkind J.S. (2020). G Protein-Coupled receptors and heterotrimeric G proteins as cancer drivers. FEBS Lett..

[12] Gutkind J.S., Novotny E.A., Brann M.R., Robbins K.C. (1991). Muscarinic acetylcholine receptor subtypes as agonist-dependent oncogenes. Proc. Natl. Acad. Sci. USA.

[13] Kalinec G., Nazarali A.J., Hermouet S., Xu N., Gutkind J.S. (1992). Mutated alpha subunit of the Gq protein induces malignant transformation in NIH 3T3 cells. Mol. Cell Biol..

[14] Feng X., Arang N., Rigiracciolo D.C., Lee J.S., Yeerna H., Wang Z., Lubrano S., Kishore A., Pachter J.A., König G.M. (2019). A Platform of Synthetic Lethal Gene Interaction Networks Reveals that the GNAQ Uveal Melanoma Oncogene Controls the Hippo Pathway through FAK. Cancer Cell.

[15] Feng X., Degese M.S., Iglesias-Bartolome R., Vaque J.P., Molinolo A.A., Rodrigues M., Zaidi M.R., Ksander B.R., Merlino G., Sodhi A. (2014). Hippo-independent activation of YAP by the GNAQ uveal melanoma oncogene through a trio-regulated rho GTPase signaling circuitry. Cancer Cell.

[16] Yu F.X., Luo J., Mo J.S., Liu G., Kim Y.C., Meng Z., Zhao L., Peyman G., Ouyang H., Jiang W. (2014). Mutant Gq/11 promote uveal melanoma tumorigenesis by activating YAP. Cancer Cell.

[17] Chen X., Wu Q., Depeille P., Chen P., Thornton S., Kalirai H., Coupland S.E., Roose J.P., Bastian B.C. (2017). RasGRP3 Mediates MAPK Pathway Activation in GNAQ Mutant Uveal Melanoma. Cancer Cell.

[18] Griner E.M., Kazanietz M.G. (2007). Protein kinase C and other diacylglycerol effectors in cancer. Nat. Rev. Cancer.

[19] Falchook G.S., Lewis K.D., Infante J.R., Gordon M.S., Vogelzang N.J., DeMarini D.J., Sun P., Moy C., Szabo S.A., Roadcap L.T. (2012). Activity of the oral MEK inhibitor trametinib in patients with advanced melanoma: a phase 1 dose-escalation trial. Lancet Oncol..

[20] Carvajal R.D., Sosman J.A., Quevedo J.F., Milhem M.M., Joshua A.M., Kudchadkar R.R., Linette G.P., Gajewski T.F., Lutzky J., Lawson D.H. (2014). Effect of selumetinib vs chemotherapy on progression-free survival in uveal melanoma: a randomized clinical trial. JAMA.

[21] Carvajal R.D., Piperno-Neumann S., Kapiteijn E., Chapman P.B., Frank S., Joshua A.M., Piulats J.M., Wolter P., Cocquyt V., Chmielowski B. (2018). Selumetinib in Combination With Dacarbazine in Patients With Metastatic Uveal Melanoma: A Phase III, Multicenter, Randomized Trial (SUMIT). J. Clin. Oncol..

[22] Vaqué J.P., Dorsam R.T., Feng X., Iglesias-Bartolome R., Forsthoefel D.J., Chen Q., Debant A., Seeger M.A., Ksander B.R., Teramoto H., Gutkind J.S. (2013). A genome-wide RNAi screen reveals a Trio-regulated Rho GTPase circuitry transducing mitogenic signals initiated by G protein-coupled receptors. Mol. Cell.

[23] Arang N., Lubrano S., Rigiracciolo D.C., Nachmanson D., Lippman S.M., Mali P., Harismendy O., Gutkind J.S. (2023). Whole-genome CRISPR screening identifies PI3K/AKT as a downstream component of the oncogenic GNAQ-Focal Adhesion Kinase signaling circuitry. J. Biol. Chem..

[24] Annala S., Feng X., Shridhar N., Eryilmaz F., Patt J., Yang J., Pfeil E.M., Cervantes-Villagrana R.D., Inoue A., Häberlein F. (2019). Direct targeting of Galphaq and Galpha11 oncoproteins in cancer cells. Sci. Signal..

[25] Schrage R., Schmitz A.L., Gaffal E., Annala S., Kehraus S., Wenzel D., Büllesbach K.M., Bald T., Inoue A., Shinjo Y. (2015). The experimental power of FR900359 to study Gq-regulated biological processes. Nat. Commun..

[26] Liu A.W., Wei A.Z., Maniar A.B., Carvajal R.D. (2022). Tebentafusp in advanced uveal melanoma: proof of principle for the efficacy of T-cell receptor therapeutics and bispecifics in solid tumors. Expet Opin. Biol. Ther..

[27] Middleton M.R., McAlpine C., Woodcock V.K., Corrie P., Infante J.R., Steven N.M., Evans T.R.J., Anthoney A., Shoushtari A.N., Hamid O. (2020). Tebentafusp, A TCR/Anti-CD3 Bispecific Fusion Protein Targeting gp100, Potently Activated Antitumor Immune Responses in Patients with Metastatic Melanoma. Clin. Cancer Res..

[28] Nathan P., Hassel J.C., Rutkowski P., Baurain J.F., Butler M.O., Schlaak M., Sullivan R.J., Ochsenreither S., Dummer R., Kirkwood J.M. (2021). Overall Survival Benefit with Tebentafusp in Metastatic Uveal Melanoma. N. Engl. J. Med..

[29] Haley B., Roudnicky F. (2020). Functional Genomics for Cancer Drug Target Discovery. Cancer Cell.

[30] Tsherniak A., Vazquez F., Montgomery P.G., Weir B.A., Kryukov G., Cowley G.S., Gill S., Harrington W.F., Pantel S., Krill-Burger J.M. (2017). Defining a Cancer Dependency Map. Cell.

[31] Lin G.L., Wilson K.M., Ceribelli M., Stanton B.Z., Woo P.J., Kreimer S., Qin E.Y., Zhang X., Lennon J., Nagaraja S. (2019). Therapeutic strategies for diffuse midline glioma from high-throughput combination drug screening. Sci. Transl. Med..

[32] Rozengurt E. (2007). Mitogenic signaling pathways induced by G protein-coupled receptors. J. Cell. Physiol..

[33] Hubbard K.B., Hepler J.R. (2006). Cell signalling diversity of the Gqalpha family of heterotrimeric G proteins. Cell. Signal..

[34] Kapiteijn E., Carlino M., Boni V., Loirat D., Speetjens F., Park J., Calvo E., Carvajal R., Nyakas M., Gonzalez-Maffe J. (2019). Abstract CT068: A Phase I trial of LXS196, a novel PKC inhibitor for metastatic uveal melanoma. Cancer Res..

[35] Piperno-Neumann S., Larkin J., Carvajal R.D., Luke J.J., Schwartz G.K., Hodi F.S., Sablin M.P., Shoushtari A.N., Szpakowski S., Chowdhury N.R. (2020). Genomic Profiling of Metastatic Uveal Melanoma and Clinical Results of a Phase I Study of the Protein Kinase C Inhibitor AEB071. Mol. Cancer Therapeut..

[36] Gschwendt M., Dieterich S., Rennecke J., Kittstein W., Mueller H.J., Johannes F.J. (1996). Inhibition of protein kinase C mu by various inhibitors. Differentiation from protein kinase c isoenzymes. FEBS Lett..

[37] Walsh C., Tanjoni I., Uryu S., Tomar A., Nam J.O., Luo H., Phillips A., Patel N., Kwok C., McMahon G. (2010). Oral delivery of PND-1186 FAK inhibitor decreases tumor growth and spontaneous breast to lung metastasis in pre-clinical models. Cancer Biol. Ther..

[38] Bain J., Plater L., Elliott M., Shpiro N., Hastie C.J., McLauchlan H., Klevernic I., Arthur J.S.C., Alessi D.R., Cohen P. (2007). The selectivity of protein kinase inhibitors: a further update. Biochem. J..

[39] Hastie C.J., McLauchlan H.J., Cohen P. (2006). Assay of protein kinases using radiolabeled ATP: a protocol. Nat. Protoc..

[40] Mukai H. (2003). The structure and function of PKN, a protein kinase having a catalytic domain homologous to that of PKC. J. Biochem..

[41] Vincent S., Settleman J. (1997). The PRK2 kinase is a potential effector target of both Rho and Rac GTPases and regulates actin cytoskeletal organization. Mol. Cell Biol..

[42] Flynn P., Mellor H., Palmer R., Panayotou G., Parker P.J. (1998). Multiple interactions of PRK1 with RhoA. Functional assignment of the Hr1 repeat motif. J. Biol. Chem..

[43] Maesaki R., Ihara K., Shimizu T., Kuroda S., Kaibuchi K., Hakoshima T. (1999). The structural basis of Rho effector recognition revealed by the crystal structure of human RhoA complexed with the effector domain of PKN/PRK1. Mol. Cell.

[44] Marinissen M.J., Chiariello M., Gutkind J.S. (2001). Regulation of gene expression by the small GTPase Rho through the ERK6 (p38 gamma) MAP kinase pathway. Genes Dev..

[45] Quétier I., Marshall J.J.T., Spencer-Dene B., Lachmann S., Casamassima A., Franco C., Escuin S., Worrall J.T., Baskaran P., Rajeeve V. (2016). Knockout of the PKN Family of Rho Effector Kinases Reveals a Non-redundant Role for PKN2 in Developmental Mesoderm Expansion. Cell Rep..

[46] Reid T., Furuyashiki T., Ishizaki T., Watanabe G., Watanabe N., Fujisawa K., Morii N., Madaule P., Narumiya S. (1996). Rhotekin, a new putative target for Rho bearing homology to a serine/threonine kinase, PKN, and rhophilin in the rho-binding domain. J. Biol. Chem..

[47] Avruch J., Zhou D., Fitamant J., Bardeesy N., Mou F., Barrufet L.R. (2012). Protein kinases of the Hippo pathway: regulation and substrates. Semin. Cell Dev. Biol..

[48] Meng Z., Moroishi T., Guan K.L. (2016). Mechanisms of Hippo pathway regulation. Genes Dev..

[49] Pérez-Guijarro E., Yang H.H., Araya R.E., El Meskini R., Michael H.T., Vodnala S.K., Marie K.L., Smith C., Chin S., Lam K.C. (2020). Multimodel preclinical platform predicts clinical response of melanoma to immunotherapy. Nat. Med..

[50] Rodrigues A., Cosman R., Joshua A.M. (2023). LXS196 for Metastatic Uveal Melanoma - finally some progress. Br. J. Cancer.

[51] Troutman S., Moleirinho S., Kota S., Nettles K., Fallahi M., Johnson G.L., Kissil J.L. (2016). Crizotinib inhibits NF2-associated schwannoma through inhibition of focal adhesion kinase 1. Oncotarget.

[52] Eisenhauer E.A., Therasse P., Bogaerts J., Schwartz L.H., Sargent D., Ford R., Dancey J., Arbuck S., Gwyther S., Mooney M. (2009). New response evaluation criteria in solid tumours: revised RECIST guideline (version 1.1). Eur. J. Cancer.

[53] Paradis J.S., Acosta M., Saddawi-Konefka R., Kishore A., Gomes F., Arang N., Tiago M., Coma S., Lubrano S., Wu X. (2021). Synthetic Lethal Screens Reveal Cotargeting FAK and MEK as a Multimodal Precision Therapy for GNAQ-Driven Uveal Melanoma. Clin. Cancer Res..

[54] Ngeow K.C., Friedrichsen H.J., Li L., Zeng Z., Andrews S., Volpon L., Brunsdon H., Berridge G., Picaud S., Fischer R. (2018). BRAF/MAPK and GSK3 signaling converges to control MITF nuclear export. Proc. Natl. Acad. Sci. USA.

[55] Eroglu Z., Chen Y.A., Gibney G.T., Weber J.S., Kudchadkar R.R., Khushalani N.I., Markowitz J., Brohl A.S., Tetteh L.F., Ramadan H. (2018). Combined BRAF and HSP90 Inhibition in Patients with Unresectable BRAF (V600E)-Mutant Melanoma. Clin. Cancer Res..

[56] Landreville S., Agapova O.A., Matatall K.A., Kneass Z.T., Onken M.D., Lee R.S., Bowcock A.M., Harbour J.W. (2012). Histone deacetylase inhibitors induce growth arrest and differentiation in uveal melanoma. Clin. Cancer Res..

[57] Faião-Flores F., Emmons M.F., Durante M.A., Kinose F., Saha B., Fang B., Koomen J.M., Chellappan S.P., Maria-Engler S.S., Rix U. (2019). HDAC Inhibition Enhances the In Vivo Efficacy of MEK Inhibitor Therapy in Uveal Melanoma. Clin. Cancer Res..

[58] Ambrosini G., Sawle A.D., Musi E., Schwartz G.K. (2015). BRD4-targeted therapy induces Myc-independent cytotoxicity in Gnaq/11-mutatant uveal melanoma cells. Oncotarget.

[59] Chua V., Aplin A.E. (2018). Novel therapeutic strategies and targets in advanced uveal melanoma. Curr. Opin. Oncol..

[60] Bailey F.P., Clarke K., Kalirai H., Kenyani J., Shahidipour H., Falciani F., Coulson J.M., Sacco J.J., Coupland S.E., Eyers P.A. (2018). Kinome-wide transcriptional profiling of uveal melanoma reveals new vulnerabilities to targeted therapeutics. Pigment Cell Melanoma Res..

[61] Mukherjee N., Dart C.R., Amato C.M., Honig-Frand A., Lambert J.R., Lambert K.A., Robinson W.A., Tobin R.P., McCarter M.D., Couts K.L. (2022). Expression Differences in BCL2 Family Members between Uveal and Cutaneous Melanomas Account for Varying Sensitivity to BH3 Mimetics. J. Invest. Dermatol..

[62] Sun Y., Han J., Wang Z., Li X., Sun Y., Hu Z. (2020). Safety and Efficacy of Bromodomain and Extra-Terminal Inhibitors for the Treatment of Hematological Malignancies and Solid Tumors: A Systematic Study of Clinical Trials. Front. Pharmacol..

[63] Kotschy A., Szlavik Z., Murray J., Davidson J., Maragno A.L., Le Toumelin-Braizat G., Chanrion M., Kelly G.L., Gong J.N., Moujalled D.M. (2016). The MCL1 inhibitor S63845 is tolerable and effective in diverse cancer models. Nature.

[64] Berginski M.E., Moret N., Liu C., Goldfarb D., Sorger P.K., Gomez S.M. (2021). The Dark Kinase Knowledgebase: an online compendium of knowledge and experimental results of understudied kinases. Nucleic Acids Res..

[65] Ma J., Weng L., Bastian B.C., Chen X. (2021). Functional characterization of uveal melanoma oncogenes. Oncogene.

[66] Collazos A., Michael N., Whelan R.D.H., Kelly G., Mellor H., Pang L.C.H., Totty N., Parker P.J. (2011). Site recognition and substrate screens for PKN family proteins. Biochem. J..

[67] Johnson J.L., Yaron T.M., Huntsman E.M., Kerelsky A., Song J., Regev A., Lin T.Y., Liberatore K., Cizin D.M., Cohen B.M. (2023). An atlas of substrate specificities for the human serine/threonine kinome. Nature.

[68] Kapiteijn E., Carlino M., Boni V., Loirat D., Speetjens F., Park J., Calvo E., Carvajal R., Nyakas M., Gonzalez-Maffe J. (2019). Abstract CT068: A Phase I trial of LXS196, a novel PKC inhibitor for metastatic uveal melanoma. Cancer Res..

[69] Park J.J., Stewart A., Irvine M., Pedersen B., Ming Z., Carlino M.S., Diefenbach R.J., Rizos H. (2022). Protein kinase inhibitor responses in uveal melanoma reflects a diminished dependency on PKC-MAPK signaling. Cancer Gene Ther..

[70] Rino S., Seedor M.O., Gutkind A.J.S., Aplin A.A.E., Terai A.M., Sharpe-Mills A.E., Klose A.H., Mastrangelo A.M.J., Sato A.T. (2021). Clinical trial in progress: Phase II trial of defactinib (VS-6063) combined with VS-6766 (CH5126766) in patients with metastatic uveal melanoma. J. Clin. Oncol..

[71] (2022).

[72] (2022).

[73] Tarin M., Némati F., Decaudin D., Canbezdi C., Marande B., Silva L., Derrien H., Jochemsen A.G., Gardrat S., Piperno-Neumann S. (2023). FAK Inhibitor-Based Combinations with MEK or PKC Inhibitors Trigger Synergistic Antitumor Effects in Uveal Melanoma. Cancers.

[74] Lietman C.D., McKean M. (2022). Targeting GNAQ/11 through PKC inhibition in uveal melanoma. Cancer Gene Ther..

[75] Metz K.S., Deoudes E.M., Berginski M.E., Jimenez-Ruiz I., Aksoy B.A., Hammerbacher J., Gomez S.M., Phanstiel D.H. (2018). Coral: Clear and Customizable Visualization of Human Kinome Data. Cell Syst..

[76] Perez-Riverol Y., Bai J., Bandla C., García-Seisdedos D., Hewapathirana S., Kamatchinathan S., Kundu D.J., Prakash A., Frericks-Zipper A., Eisenacher M. (2022). The PRIDE database resources in 2022: a hub for mass spectrometry-based proteomics evidences. Nucleic Acids Res..

[77] Inglese J., Auld D.S., Jadhav A., Johnson R.L., Simeonov A., Yasgar A., Zheng W., Austin C.P. (2006). Quantitative high-throughput screening: a titration-based approach that efficiently identifies biological activities in large chemical libraries. Proc. Natl. Acad. Sci. USA.

[78] Swaney D.L., Ramms D.J., Wang Z., Park J., Goto Y., Soucheray M., Bhola N., Kim K., Zheng F., Zeng Y. (2021). A protein network map of head and neck cancer reveals PIK3CA mutant drug sensitivity. Science.

[79] Cox J., Mann M. (2008). MaxQuant enables high peptide identification rates, individualized p.p.b.-range mass accuracies and proteome-wide protein quantification. Nat. Biotechnol..

[80] Teo G., Liu G., Zhang J., Nesvizhskii A.I., Gingras A.C., Choi H. (2014). SAINTexpress: improvements and additional features in Significance Analysis of INTeractome software. J. Proteomics.

[81] Vizcaíno J.A., Deutsch E.W., Wang R., Csordas A., Reisinger F., Ríos D., Dianes J.A., Sun Z., Farrah T., Bandeira N. (2014). ProteomeXchange provides globally coordinated proteomics data submission and dissemination. Nat. Biotechnol..

[82] Perez-Riverol Y., Csordas A., Bai J., Bernal-Llinares M., Hewapathirana S., Kundu D.J., Inuganti A., Griss J., Mayer G., Eisenacher M. (2019). The PRIDE database and related tools and resources in 2019: improving support for quantification data. Nucleic Acids Res..

[83] Wang Z., Feng X., Molinolo A.A., Martin D., Vitale-Cross L., Nohata N., Ando M., Wahba A., Amornphimoltham P., Wu X. (2019). 4E-BP1 Is a Tumor Suppressor Protein Reactivated by mTOR Inhibition in Head and Neck Cancer. Cancer Res..

[84] Iglesias-Bartolome R., Torres D., Marone R., Feng X., Martin D., Simaan M., Chen M., Weinstein L.S., Taylor S.S., Molinolo A.A., Gutkind J.S. (2015). Inactivation of a Galpha(s)-PKA tumour suppressor pathway in skin stem cells initiates basal-cell carcinogenesis. Nat. Cell Biol..

[85] Schindelin J., Arganda-Carreras I., Frise E., Kaynig V., Longair M., Pietzsch T., Preibisch S., Rueden C., Saalfeld S., Schmid B. (2012). Fiji: an open-source platform for biological-image analysis. Nat. Methods.

