# Supplementary material for: High-throughput chemogenetic drug screening reveals PKC-RhoA/PKN as a targetable signaling vulnerability in GNAQ-driven uveal melanoma
Source: Cell Rep Med. 2023 Oct 18;4(11):101244. doi: 10.1016/j.xcrm.2023.101244 (PMC10694608; doi:10.1016/j.xcrm.2023.101244)
Supplement: Document S1. Figures S1−S5 and Tables S2 and S3 [file mmc1.pdf]

Cell Reports Medicine, Volume *4*

Supplemental information

High-throughput chemogenetic drug screening reveals PKC-RhoA/PKN as a targetable signaling vulnerability in *GNAQ*-driven uveal melanoma

Nadia Arang, Simone Lubrano, Michele Ceribelli, Damiano C. Rigiracciolo, Robert Saddawi-Konefka, Farhoud Faraji, Sydney I. Ramirez, Daehwan Kim, Frances A. Tosto, Erica Stevenson, Yuan Zhou, Zhiyong Wang, Julius Bogomolovas, Alfredo A. Molinolo, Danielle L. Swaney, Nevan J. Krogan, Jing Yang, Silvia Coma, Jonathan A. Pachter, Andrew E. Aplin, Dario R. Alessi, Craig J. Thomas, and J. Silvio Gutkind

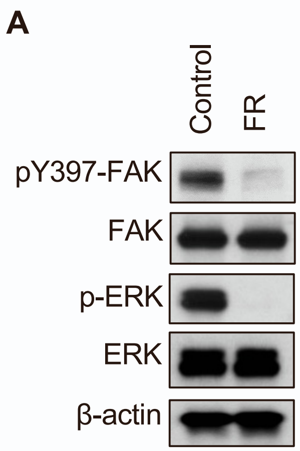

Figure S1, related to Fig 2: Impact of Gαq inhibition on FAK and ERK signaling in UM. Changes in phosphorylation of FAK and ERK in UM cells in response to 500nM FR900359 treatment for 2hrs in 92.1 UM cells.

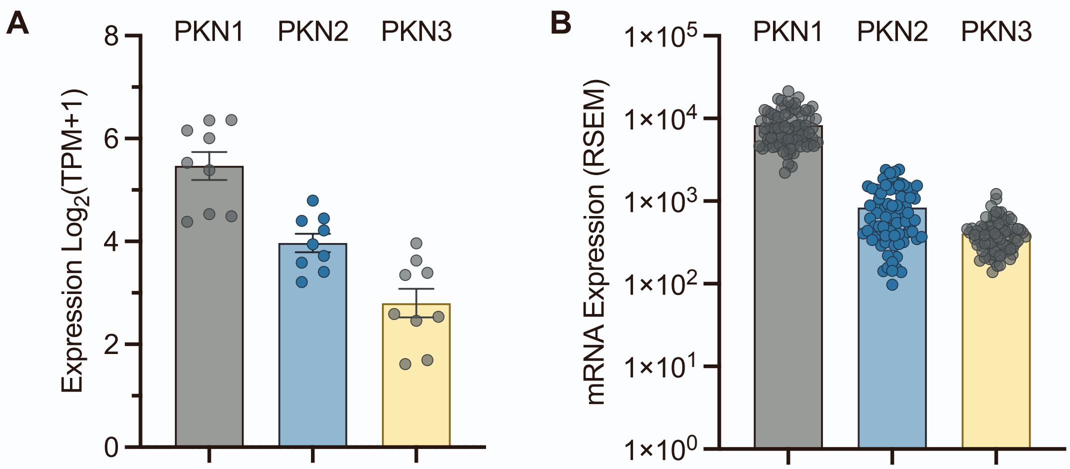

Figure S2, related to Fig 3: **mRNA expression of PKN isoforms in UM cells.** PKN expression in UM cell lines from DepMap 22Q2 Expression Public database (**A**) and TCGA Uveal Melanoma cohort patient samples (**B**)

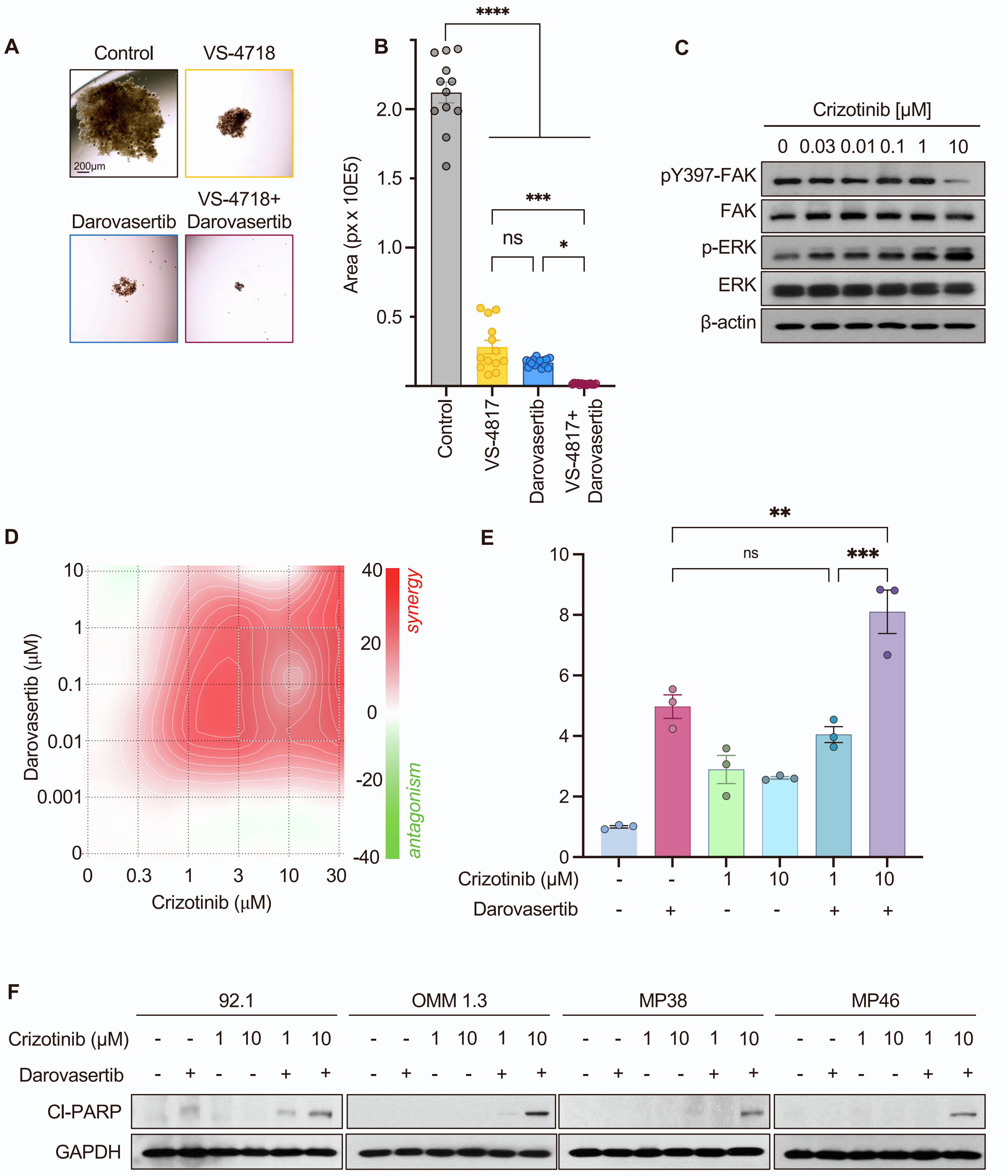
A
Control
VS-4718
200μm
Darovasertib
VS-4718+
Darovasertib
B
****
***
ns
*
Area (px x 10E5)
2.0
1.5
1.0
0.5
Control
VS-4817
Darovasertib
VS-4817+
Darovasertib
C
Crizotinib [μM]
0
0.03
0.01
0.1
1
10
pY397-FAK
FAK
p-ERK
ERK
β-actin
D
Darovasertib (μM)
10
1
0.1
0.01
0.001
0
0.3
3
30
Crizotinib (μM)
40
20
-20
-40
synergy
antagonism
E
**
ns
***
10
8
6
4
2
Crizotinib (μM)
Darovasertib
F
92.1
OMM 1.3
MP38
MP46
Crizotinib (μM)
Darovasertib
Cl-PARP
GAPDH

Figure S3, related to Figure 5: Assessment of darovasertib-centered drug combinations in UM. Inhibition of UM proliferation in 3D growth conditions in response to 1μM VS-4718, 1μM darovasertib, or 1μM VS-4718+1μM darovasertib in 92.1 UM cells for 20 days. Representative images in (**A**) and quantification of growth area in (**B**). **C**) Effect of crizotinib on phosphorylation of FAK in 92.1 UM cells. (**D**) Assessment of synergy in UM cells treated with darovasertib and crizotinib. CI was determined using HSA method. **E**) Induction of apoptosis measured by CapsaseGlo 3/7 with 1μM darovasertib and indicated doses of crizotinib for 24 hrs. (mean ± SEM, n = 3). **F**) Immunoblot showing cleaved-PARP levels in response to treatment with 1μM darovasertib and indicated doses of crizotinib for 24 hrs in UM cells.

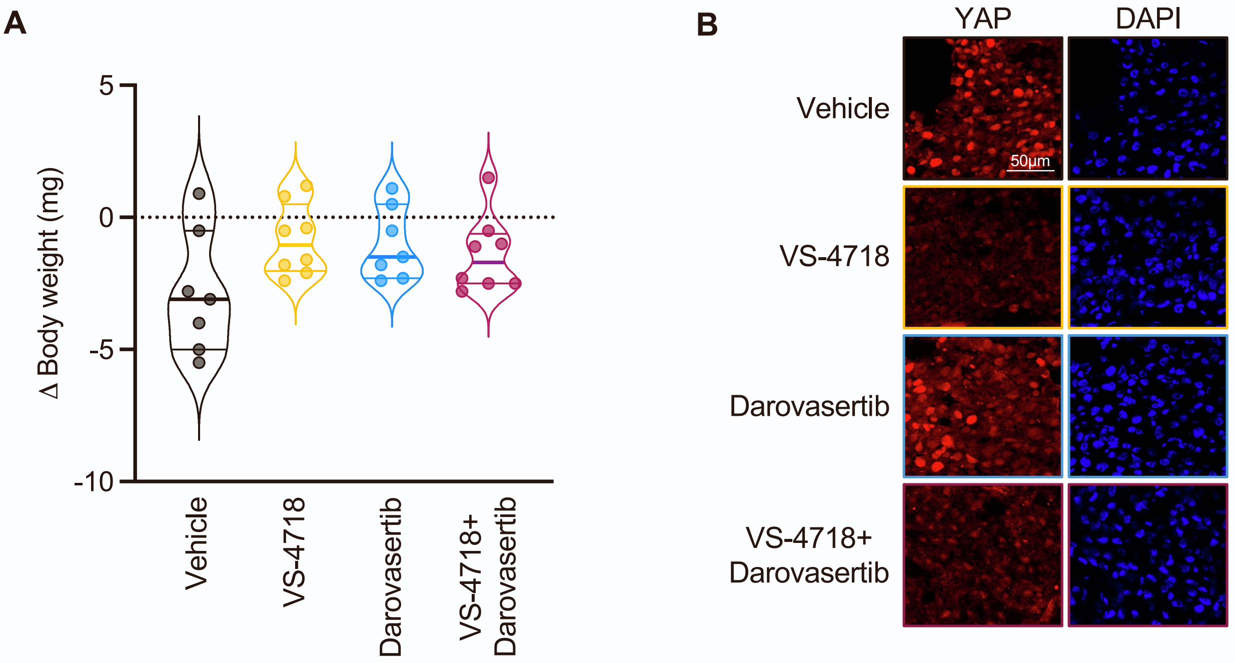

Figure S4, related to Figure 6: Tolerability of darovasertib+VS-4718 and impact on downstream signaling. A) Violin plot depicting changes in body weight of mice between day 0 and day 25 with respect to the indicated treatment groups from Fig. 6A. Thick center line represents mean with upper and lower quartiles. **B)** Single channel images of YAP and DAPI staining in UM xenograft tumors treated with vehicle (control), VS-4718 50mg/kg BID PO, darovasertib 50mg/kg BID PO, or VS-4718 50mg/kg BID PO + darovasertib 50mg/kg BID PO.

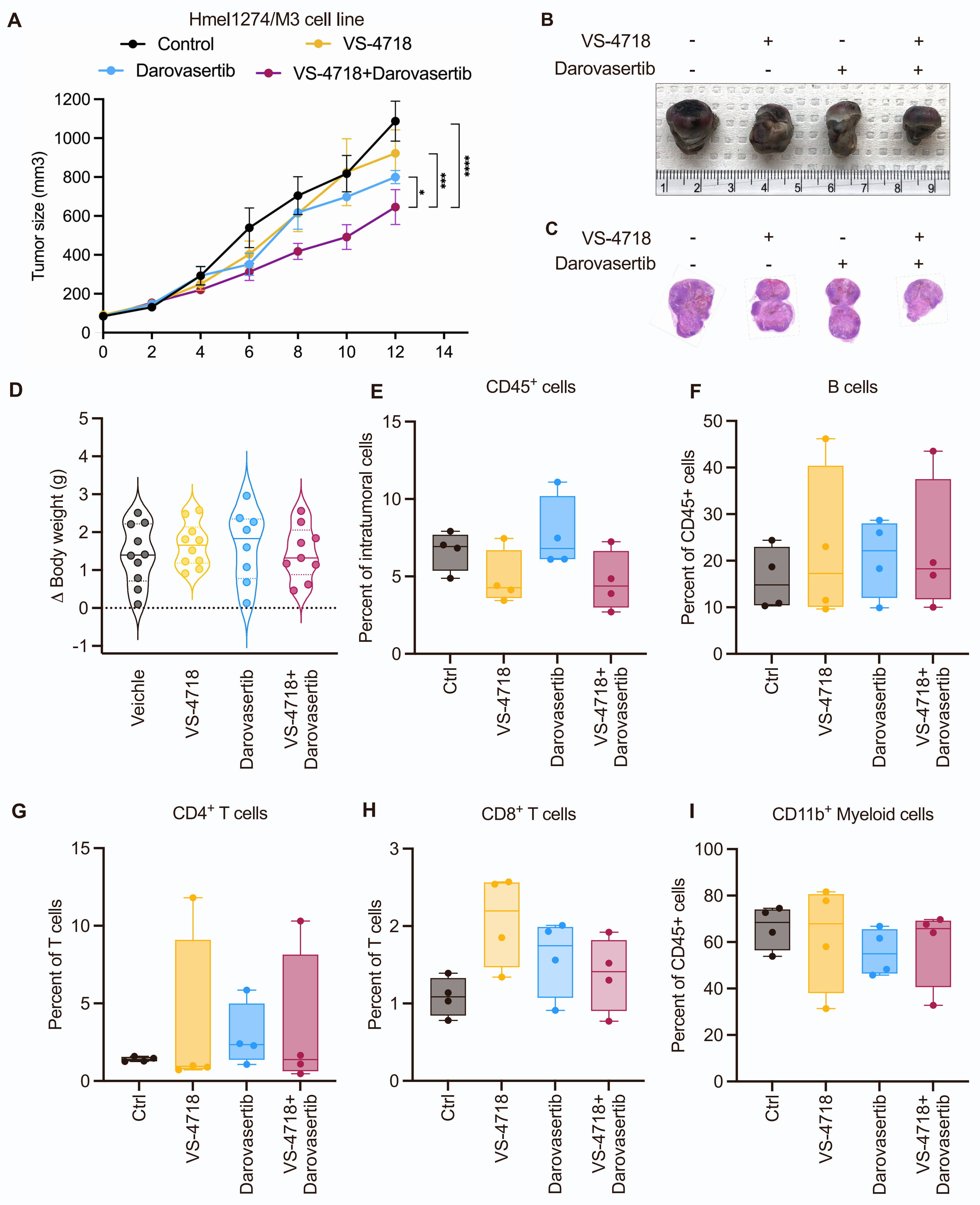

A
Hmel1274/M3 cell line
Control
VS-4718
Darovasertib
VS-4718+Darovasertib
Tumor size (mm3)
*
***
****
B
VS-4718
Darovasertib
C
VS-4718
Darovasertib
D
Δ Body weight (g)
Veichle
VS-4718
Darovasertib
VS-4718+ Darovasertib
E
CD45+ cells
Percent of intratumoral cells
Ctrl
VS-4718
Darovasertib
VS-4718+ Darovasertib
F
B cells
Percent of CD45+ cells
G
CD4+ T cells
Percent of T cells
H
CD8+ T cells
Percent of T cells
I
CD11b+ Myeloid cells
Percent of CD45+ cells

Figure S5, related to Figure 6: Efficacy of darovasertib + VS-4718 in an immunocompetent syngeneic model of UM. A) Hmel1274/M3 syngeneic melanoma model tumor growth kinetics in C57BL/6 mice treated with vehicle (control), VS-4718 50mg/kg BID PO, darovasertib 50mg/kg BID PO, or combination of VS-4718 50mg/kg BID PO + darovasertib 50mg/kg BID PO. Data are mean ± SEM (>5 mice/group). **B)** Image of tumors at termination of treatment. **C**) H&E staining of representative xenograft tumor sections from (**A**) after 12 days of treatment. **D**) Violin plot depicting changes in body weight of mice between day 0 and day 12 with respect to the indicated treatment groups from Fig. S5A. Thick center line represents mean with upper and lower quartiles. **E-I)** Analysis of indicated intratumoral immune cells in response to treatment from tumors in (**A**)

Drug set	Enriched Subtype	Size	Enrich Score (ES)	Normalized ES (NES)	NOM p-val	FDR q-val	FWER p-val
TUBB	UVM	37	-0.7393	-2.33548	0	0	0
BRD2-4	UVM	17	-0.88968	-2.29194	0	0	0
HDAC1	UVM	18	-0.75078	-2.02309	0	0.000466	0.002
BRD4	UVM	13	-0.87078	-2.10563	0	0.000621	0.002
PRKCs	UVM	8	-0.90261	-1.92185	0	0.001818	0.009
HDAC6	UVM	10	-0.79031	-1.78519	0	0.016091	0.096
CDK1	UVM	13	-0.71079	-1.73603	0.002028	0.023504	0.203
BRD2	UVM	4	-0.95931	-1.73674	0	0.0262	0.201
MCL1	UVM	4	-0.95411	-1.69547	0	0.036792	0.324
XPO1	UVM	5	-0.90122	-1.67745	0.001923	0.043458	0.388
CHEK1	UVM	13	-0.6936	-1.65213	0.010965	0.057668	0.504
EP300	UVM	6	-0.81932	-1.61649	0.003953	0.081759	0.666
CDK4	UVM	11	-0.68555	-1.61076	0.026374	0.081844	0.691

Table S2. Target-level enrichment scores and significance values for drug sets enriched in UM subtype, related to Fig 1.

Drug set	Enriched Subtype	Size	Enrich Score (ES)	Normalized ES (NES)	NOM p-val	FDR q-val	FWER p-val
BRAF	SKCM	18	0.893223	2.239835	0	0	0
HSP90AB1	SKCM	18	0.832443	2.142556	0	0	0
MAP2K1	SKCM	16	0.864533	2.071136	0	0	0
GSK3B	SKCM	10	0.852684	1.855847	0	0.015604	0.059
MAPK3	SKCM	6	0.887301	1.723525	0.003984	0.068169	0.33
LCK	SKCM	4	0.936616	1.727005	0.006186	0.076648	0.313
NAMPT	SKCM	4	0.978784	1.688923	0	0.097679	0.489
PIK3CB	SKCM	13	0.70094	1.611222	0.026616	0.180722	0.863
PIK3CA	SKCM	43	0.538217	1.603144	0.010204	0.183028	0.885
AURKB	SKCM	7	0.805964	1.621923	0.015564	0.190283	0.811
ADRB2	SKCM	3	0.980832	1.629087	0.001957	0.194295	0.78
CDC7	SKCM	4	0.904913	1.612	0.010288	0.196192	0.857

Table S3. Target-level enrichment scores and significance values for drug sets enriched in SKCM subtype, related to Fig 1.
